# Developing high-affinity decoy receptors to treat multiple myeloma and diffuse large B cell lymphoma

**DOI:** 10.1084/jem.20220214

**Published:** 2022-07-26

**Authors:** Yu Rebecca Miao, Kaushik Thakkar, Can Cenik, Dadi Jiang, Kazue Mizuno, Chenjun Jia, Caiyun Grace Li, Hongjuan Zhao, Anh Diep, Yu Xu, Xin Eric Zhang, Teddy Tat Chi Yang, Michaela Liedtke, Parveen Abidi, Wing-sze Leung, Albert C. Koong, Amato J. Giaccia

**Affiliations:** 1 Department of Radiation Oncology, Stanford University, Stanford, CA; 2 Department of Molecular Biosciences, University of Texas at Austin, Austin, TX; 3 Department of Radiation Oncology, MD Anderson Cancer Center, Houston, TX; 4 ChemPartner Shanghai, Shanghai, China; 5 Department of Urology, Stanford University, Stanford, CA; 6 Department of Medicine (Hematology), Stanford University, Stanford, CA; 7 Department of Oncology, Oxford Institute for Radiation Oncology, University of Oxford, Oxford, UK

## Abstract

Disease relapse and treatment-induced immunotoxicity pose significant clinical challenges for patients with hematological cancers. Here, we reveal distinctive requirements for neutralizing TNF receptor ligands APRIL and BAFF and their receptor activity in MM and DLBCL, impacting protein translation and production in MM cells and modulating the translation efficiency of the ATM interactor (ATMIN/ACSIZ). Therapeutically, we investigated the use of BCMA decoy receptor (sBCMA-Fc) as an inhibitor of APRIL and BAFF. While wild-type sBCMA-Fc effectively blocked APRIL signaling in MM, it lacked activity in DLBCL due to its weak BAFF binding. To expand the therapeutic utility of sBCMA-Fc, we engineered an affinity-enhanced mutant sBCMA-Fc fusion molecule (sBCMA-Fc V3) 4- and 500-fold stronger in binding to APRIL and BAFF, respectively. The mutant sBCMA-Fc V3 clone significantly enhanced antitumor activity against both MM and DLBCL. Importantly, we also demonstrated an adequate toxicity profile and on-target mechanism of action in nonhuman primate studies.

## Teaser

Miao et al. demonstrate the dichotomous activities of APRIL and BAFF in MM and DLBCL, which can be safely targeted by an engineered decoy receptor designed to trap both ligands with ultra-high binding affinity.

## Introduction

B cell malignancies, in particular multiple myeloma (MM) and diffuse large B cell lymphoma (DLBCL), represent some of the most common hematological cancers worldwide (Chim et al., 2018; Durer et al., 2020; Swerdlow et al., 2016; Weber and Schmitz, 2022). While the use of treatment regimens combining chemo-cytotoxic drugs and targeted therapies has significantly improved overall survival, patients suffering from relapse/refractory diseases after standard-of-care treatment still face poor outcomes (Caimi et al., 2021; Lonial et al., 2020; Raje et al., 2019). The recent approval of chimeric antigen receptor (CAR) T cells has provided a more effective treatment for patients who failed to respond to conventional therapies, with some patients able to achieve complete remission. However, restrictive patient eligibility, immune-related adverse toxicities, and treatment relapse are challenges remain to overcome. Therefore, safe and effective targeted therapies for patients who exhaust currently available treatment options is still needed.

Two ligands of the TNF superfamily, known as a proliferation-inducing ligand (APRIL) and B cell activating factor (BAFF), are well documented as critical regulators of B cell maturation and differentiation (Bolkun et al., 2014; Chiu et al., 2007). APRIL and BAFF facilitate their diverse functions on B lymphocytes through binding to three TNF receptors, TACI (transmembrane activator and Ca^2+^ modulator interactor), B cell maturation antigen (BCMA), and BAFF receptor (BAFF-R), with various affinities (Wu et al., 2000; Yu et al., 2000). TACI and BAFF-R are present on mature B cells, whereas BCMA is almost exclusively confined to plasma cells (O’Connor et al., 2004). Differences in binding affinities between APRIL and BAFF toward TACI, BCMA, and BAFF-R in part result in highly differentiated receptor–ligand interactions to promote unique biological outcomes. For example, APRIL possesses a 1,000-fold stronger binding affinity to BCMA than BAFF, whereas the reverse is reported for BAFF–TACI and BAFF–BAFF-R interactions (Marsters et al., 2000; Schuepbach-Mallepell et al., 2015; Yu et al., 2000). This distinctive receptor–ligand binding relationship further supports the differentiated roles of APRIL and BAFF in B cell biology: APRIL-BCMA signaling is an exclusive regulator of plasma cells, and BAFF-TACI/BAFF-R activation is required for the maturation of B lymphocytes (Moreaux et al., 2007; Pelletier et al., 2003). In oncology, aberrant expression of APRIL and BAFF supports disease progression and is associated with a poor treatment outcome in MM and DLBCL. Persistent APRIL and BAFF activation promotes survival advantages in MM and DLBCL, facilitating disease progression and treatment resistance (Kuo et al., 2008; Moreaux et al., 2004). Similar to the dichotomous relationship between APRIL and BAFF in normal B cell development, APRIL-BCMA signaling is critical in MM progression, where persistent activation of BAFF-TACI/BAFF-R is critical for promoting malignant B cell growth and survival (Pham et al., 2011). Therefore, neutralizing APRIL and BAFF in B cell malignancies may offer a new treatment option for MM and DLBCL.

In this study, we investigated the therapeutic strategy of using soluble BCMA as a ligand trap for blocking APRIL-mediated signaling in MM. We also discovered a new function of BCMA signaling as a regulator of the translation of a subset of proteins, including ATMIN, a protein critical for the development of both normal and malignant B cells (Jurado et al., 2012b). Therapeutically, we developed an affinity-enhanced, soluble BCMA-based mutant Fc fusion protein, trapping both APRIL and BAFF with stronger binding affinities. This high-affinity fusion protein showed superior blockade of both APRIL/BCMA signaling and BAFF-TACI/BAFF-R signaling in MM and DLBCL models, while demonstrating little toxicity and an on-target mechanism of action in nonhuman primate studies.

## Results

### BCMA signaling activation is required for MM progression

**Figure 1.**
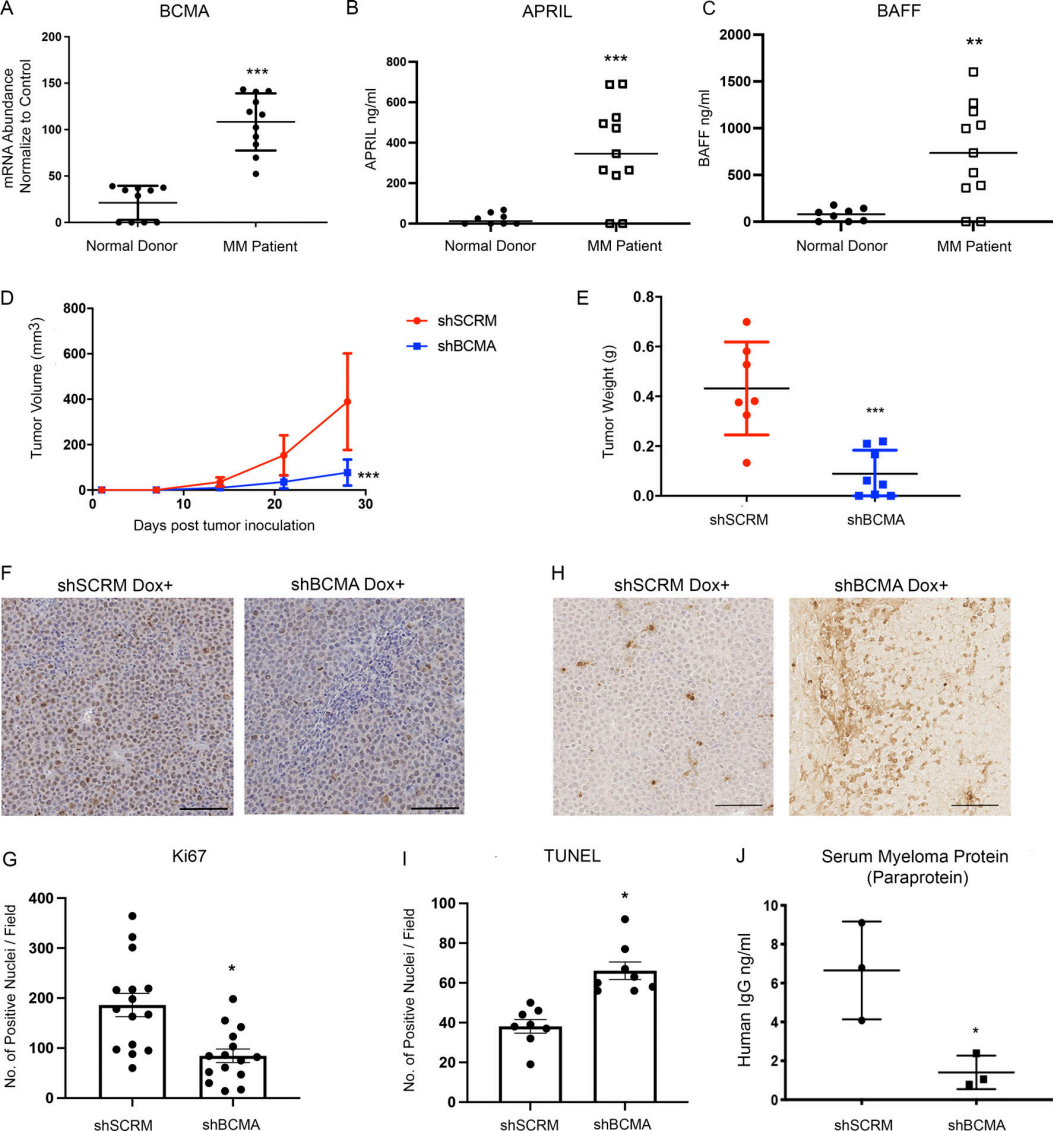
**BCMA and its ligands APRIL and BAFF are upregulated in MM and support the growth and survival of MM in vivo. (A)** BCMA mRNA transcript validated in patient myeloma cells (*n* = 11) and plasma cells harvested from healthy donors (*n* = 6); P = 0.0001. **(B)** Serum APRIL level detected in MM patients (*n* = 11) compared with healthy donors (*n* = 8) through ELISA; P = 0.001. **(C)** Serum BAFF level detected in MM patients (*n* = 11) compared with healthy donors (*n* = 8) through ELISA; P = 0.0028. **(D)** Subcutaneous tumor growth of INA-6 MM cells with stably transfected dox-inducible BCMA KO shRNA (*n* = 8) or scramble shRNA (*n* = 7) in 6-wk-old female NSG mice; P = 0.0005. **(E)** Comparing tumor weights of terminally harvested mice inoculated with dox-inducible shSCRM (*n* = 7) and shBCMA (*n* = 8) in INA-6 MM tumor cells; P = 0.0005. **(F)** Representative images of Ki67-positive cells in the harvested tumors of dox-inducible shSCRM and shBCMA, analyzed by immunohistochemical (IHC) staining. Scale bar, 50 μm. **(G)** Quantitative analysis of Ki67-positive cells in the harvested tumors of dox-inducible shSCRM and shBCMA, represented as the average number of positive nuclei per image field; P = 0.0008. **(H)** Representative images of TUNEL-positive cells in the harvested tumors of dox-inducible shSCRM and shBCMA, analyzed by IHC staining. Scale bar, 50 μm. **(I)** Quantitative analysis of TUNEL-positive cells in the harvested tumors of dox-inducible shSCRM and shBCMA, represented as the average number of positive nuclei per image field; P = 0.0002. **(J)** Total human M protein (paraprotein) detected in the serum of NSG mice inoculated with dox-inducible shSCRM and shBCMA human INA-6 MM tumor cells harvested terminally (*n* = 3); P = 0.0268. Statistical analysis was conducted using one-way ANOVA for comparing between treatment groups and repeated ANOVA for changes occurring over time. *, P < 0.05; **, P < 0.01; ***, P < 0.001.

**Figure S1.**
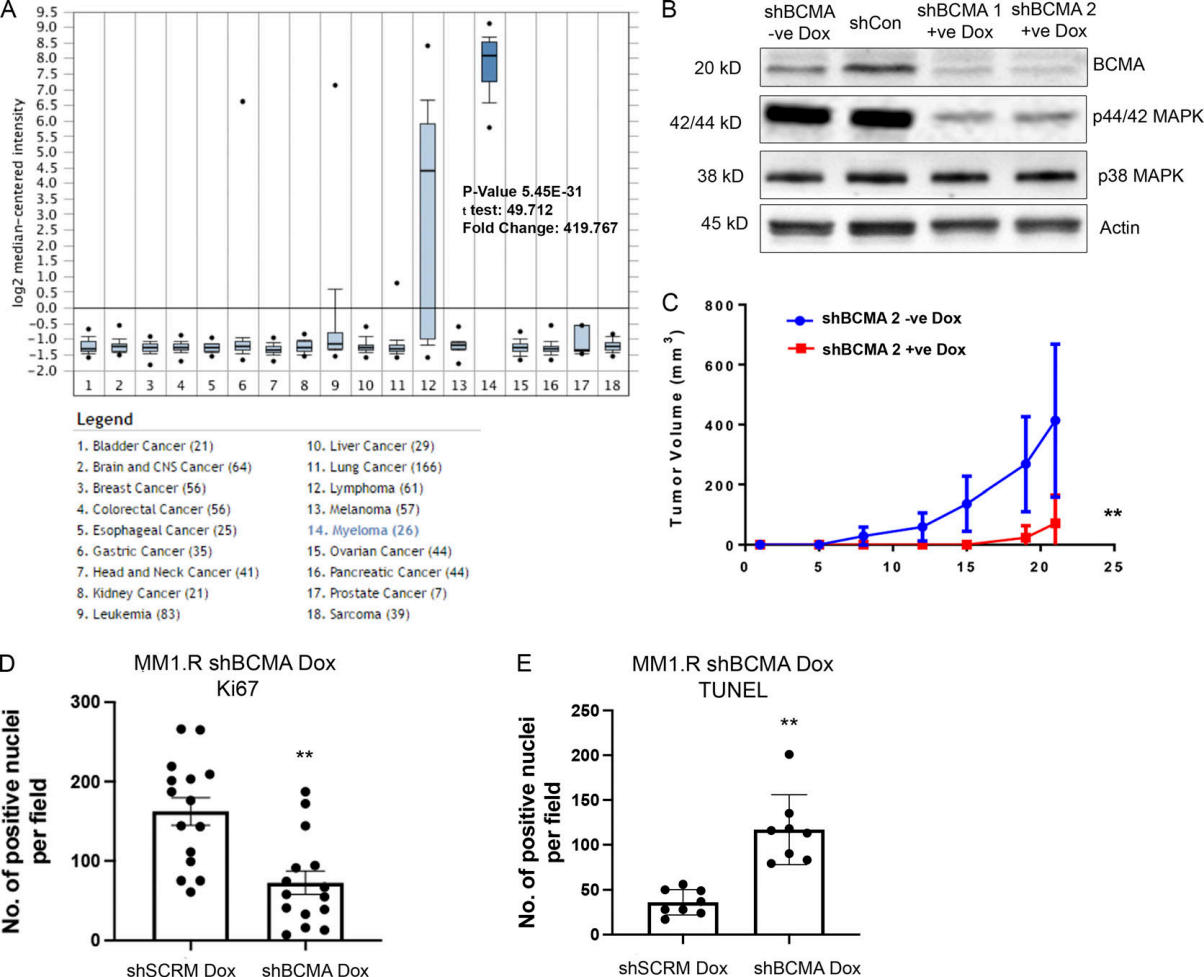
**BCMA signaling is essential for the growth and survival of MM. (A)** BCMA mRNA expression in a panel of 18 tumor cell lines queried through Oncomine. BCMA expression is significantly elevated in myeloma cell line; P = 5.45 × 10^−31^. **(B)** Western blotting analysis confirming knockdown of BCMA in MM cell line by dox-inducible shRNA. **(C)** Tumor growth kinetics in MM1.R MM cells transfected with inducible dox shBCMA, mice were given drinking water with or without dox (5 mg/ml); P = 0.007. **(D)** Quantitative analysis of Ki67-positive cells in the harvested tumors of dox-inducible shSCRM and shBCMA, represented as the average number of positive nuclei per image field; P = 0.0055. **(E)** Quantitative analysis of TUNEL-positive cells in the harvested tumors of dox-inducible shSCRM and shBCMA, represented as the average number of positive nuclei per image field; P = 0.0026. Statistical analysis was conducted using one-way ANOVA for comparing between treatment groups and repeated ANOVA for changes occurring over time. *, P < 0.05; **, P < 0.01; ***, P < 0.001. Source data are available for this figure: SourceData FS1.

We began our study by validating the functional significance of BCMA signaling in MM using genetic and biochemical approaches. Consistent with previous reports, BCMA mRNA expression was uniquely elevated on MM and B cell lymphoma and was not detectable in cancer cells other than those of a B cell lineage (Figs. 1 A and S1 A). High levels of APRIL (Fig. 1 B) and BAFF (Fig. 1 C) were detected in MM patients, also consistent with previous reports that both ligands serve as biomarkers of various B cell malignancies (Bolkun et al., 2014; Fragioudaki et al., 2012).

The dependence of MM proliferation on BCMA signaling was tested by introducing a Tet-off doxycycline (dox)-controlled BCMA stable knockdown system (dox shBCMA) into MM cell lines INA-6 and MM1.R (Fig. S1 B). INA-6 MM cells have a chromosomal translocation at t(11;14) and *NRAS* and *TP53* mutations and are dependent on IL-6 for growth and survival (Burger et al., 2001; Keats et al., 2007). MM1.R has chromosomal translocations at t(14;16) and t(8;14) and *KRAS* and *TRAF3* mutations (Greenstein et al., 2003). Genetic inhibition of BCMA with dox-inducible shBCMA led to a significant decrease in established MM tumor size in both models (Fig. 1, D and E; and Fig. S1 C), further demonstrating that BCMA signaling can act as a master regulator of MM survival and is independent of heterogeneous genetic mutations. Smaller tumor size is associated with decreased proliferation (Fig. 1, F and G; and Fig. S1 D) and increased apoptosis (Fig. 1, H and I; and Fig. S1 E) upon genetic inhibition of BCMA. Furthermore, the level of human myeloma immunoglobulin protein (paraprotein) secreted by MM tumor cells was significantly reduced in mice bearing shBCMA MM tumors, indicating decreased tumor burden (Fig. 1 J; Collier and Jackson, 1953).

### Ribosome profiling reveals distinct changes in protein translation regulated by BCMA signaling

**Figure 2.**
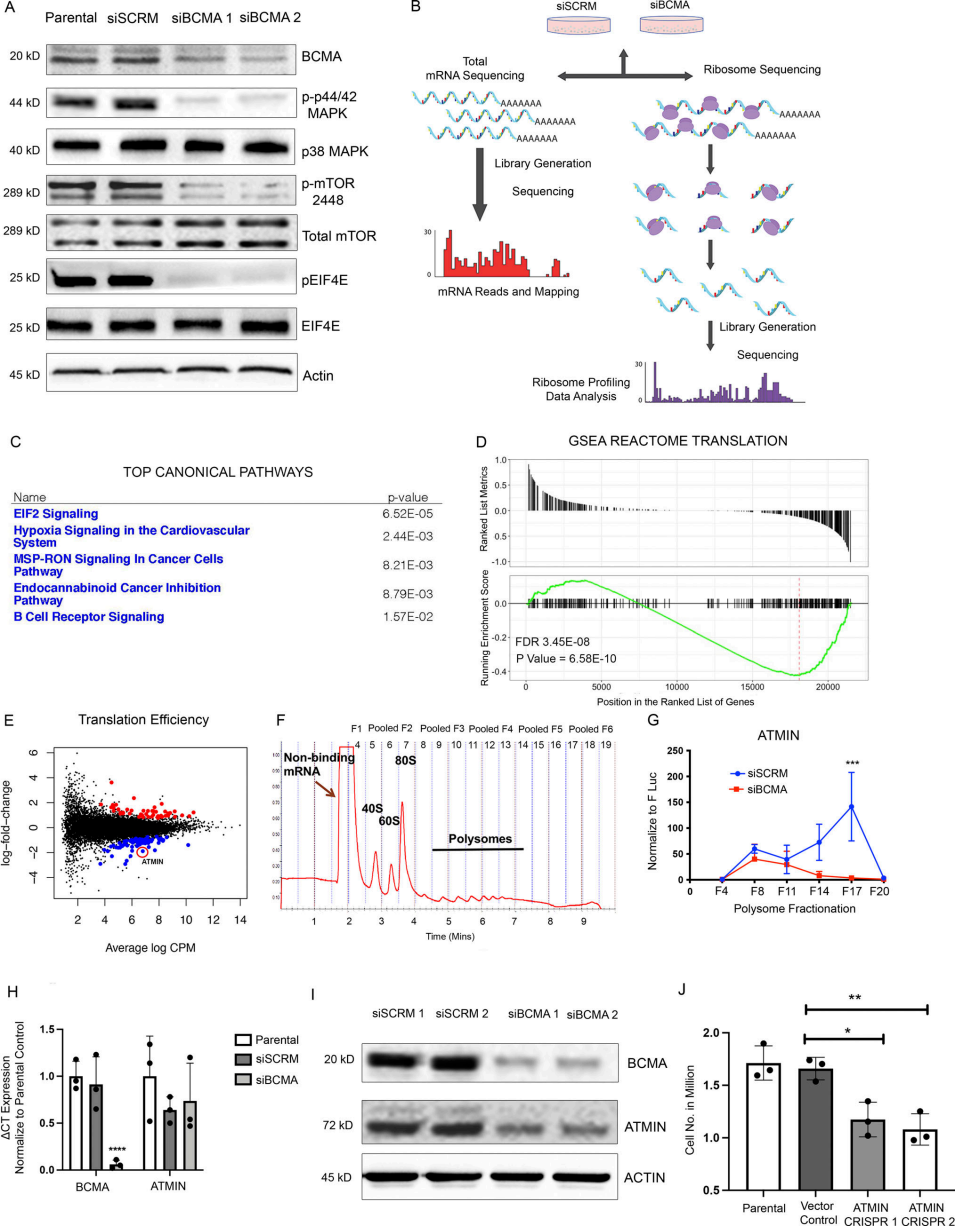
**Ribosome profiling identifies BCMA as a master regulator of protein translation machinery in MM. (A)** Western blotting analysis of changes in protein expression associated with protein translation upon genetic knockdown using siBCMA. **(B)** Schematic illustration of ribosome profiling workflow. **(C)** Ingenuity pathway analysis of RNA-seq data showing top five significantly changed canonical pathways. **(D)** GSEA REACTOME enrichment analysis showing enriched signatures in associated with protein translation. FDR, false discovery rate. **(E)** Translation efficiency analysis of MM cells upon loss of BCMA expression in MM. Significant events are colored in blue (downregulated) and red (upregulated). ATMIN highlighted with red circle. **(F)** Representative polysome profile of MM cells fractionated by ultracentrifugation through a 10–50% sucrose gradient. Fractions were pooled for mRNA analysis. **(G)** Relative abundance of ATMIN mRNA expression analyzed in each of the pooled polysome fractions comparing siSCRM with siBCMA MM cells. Each biological sample were performed in triplicate. P = 0.0228. **(H)** Total mRNA expression of BCMA (P = 0.0078) and ATMIN (NS) examined in parental, siSCRM, and siBCMA U266 cells. Each biological sample was performed in triplicate. **(I)** Western blotting analysis of ATMIN expression in siSCRM and two clones of siBCMA MM cells. **(J)** Cell growth analysis comparing ATMIN vector control with ATMIN CRISPR KO clone #1 (P = 0.0129) and with ATMIN CRISPR KO clone #2 (P = 0.0055) in U266 MM cells. Statistical analysis was conducted using *t* test and one-way ANOVA for comparing between treatment groups. *, P < 0.05; **, P < 0.01; ***, P < 0.001. Source data are available for this figure: SourceData F2.

**Figure S2.**
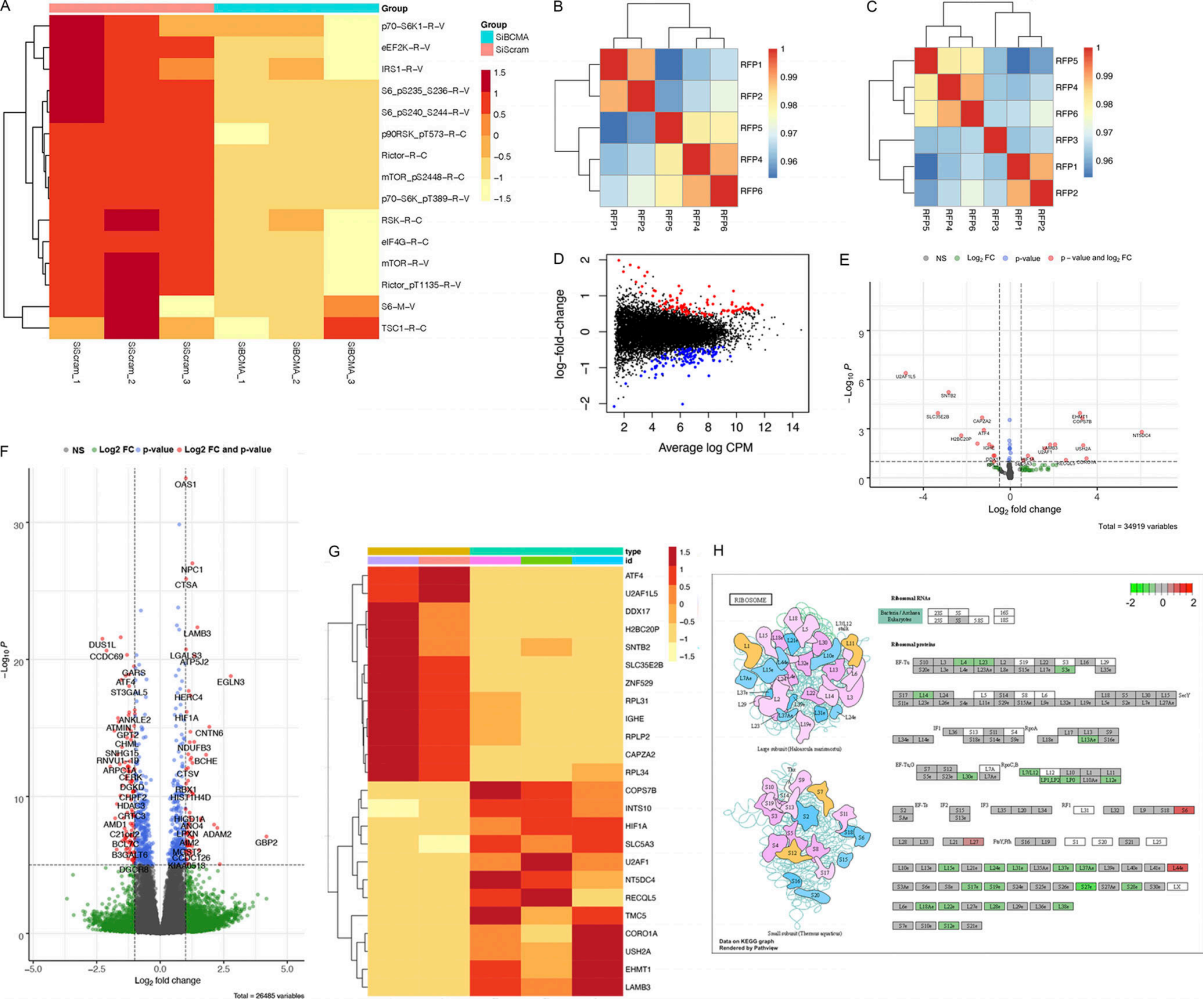
**BCMA is a critical regulator of protein translation machinery.** **(A)** Loss of BCMA-induced changes in downstream targets associated with protein translation analyzed through RPPA. **(B)** Spearman correlation analysis of ribosome profiling samples showing consistent reads across all samples with the exception of RFP3. **(C)** Spearman correlation analysis after RFP3 was removed from the dataset. **(D)** Distribution plot of changes in mRNA expression associated with genetic knockdown of BCMA. **(E)** Volcano plot analysis identifying significant changes of downstream targets within total RNA transcript associated with BCMA knockdown. Color-coded dots show statistical significance associated with log_10_Fc, P value, or both. FC, fold-change. **(F)** Volcano plot analysis identifying significant changes of downstream targets within ribosome-bound RNA associated with BCMA knockdown. Color-coded dots show statistical significance associated with identified targets categorized into log_10_Fc, P value, or both. **(G)** Representative heatmap of RPPA analysis showing subset of the significant changes in protein expression landscape associated with BCMA signaling axis. **(H)** Graphic illustration of large ribosome subunits (top) and small ribosome subunits (bottom). Each subunit shows changes in translation abundance upon BCMA loss. Decreased expression (green), no change (gray), or increased expression (red).

Upon analyzing downstream biological changes associated with BCMA signaling, we found robust changes in key regulators of protein translation and synthesis such as mTOR and the EIF family member eIF4E, suggesting a potential link between BCMA signaling and protein translation that has not been described previously (Figs. 2 A and S2 A). To investigate whether BCMA acts as a regulator of protein translation, we performed ribosome profiling on U266 MM cells with siRNA BCMA knockdown. U266 harbors some of the most frequently found genetic alternations in MM patients and is a reasonable representative of MM tumor cells (Keats et al., 2007). Ribosome profiling is a specialized analysis method that provides a quantitative measure of gene-specific translation efficiency. Technically, mRNA fragments protected by ribosomes were isolated and sequenced, and in the same population of cells, total RNA abundance was quantified by RNA sequencing (RNA-seq) for normalization (Fig. 2 B; Ingolia et al., 2011). Quality control analysis of the ribosome profiling data was performed to ensure accurate ribosome-bound RNA readouts (Fig. S2, B and C). Total mRNA obtained from RNA-seq data served as a reference for quantitating ribosome-bound mRNA–associated changes in translation efficiency (Fig. S2, D and E), and translation efficiency was analyzed from ribosome-bound mRNA normalized against total mRNA (Cenik et al., 2015). Ingenuity pathway analysis of total mRNA and GSEA reactome analysis of ribosome sequencing (Ribo-seq) data both showed enrichment of pathways and gene signatures associated with protein translation (Fig. 2, C and D). Ribo-seq data are also presented in the format of volcano plot (Fig. S2 F). Furthermore, pathways that are responsible for the synthesis of both large and small ribosome subunits were affected in BCMA-inhibited MM cells (Fig. S2 H). This finding was further confirmed by reverse-phase protein array (RPPA), which showed a decreased protein translation signature upon loss of BCMA (Fig. S2 G). Collectively, these data suggest a previously unreported role of BCMA signaling as a regulator of protein translation machinery in MM growth.

Upon further investigation, we found that the translation efficiency of ATMIN (ASCIZ) to be significantly downregulated upon loss of BCMA, while its mRNA transcription level remained unchanged (Fig. 2 E). ATMIN and its binding partner Dynein light chain have been reported to regulate the development of B cell malignancies (Jurado et al., 2012a; Jurado et al., 2012b; Leszczynska et al., 2016; Rapali et al., 2011). Initially discovered as an ATM interacting protein, ATMIN is often associated with ATM-mediated signaling and recruitment of 53BP1 upon DNA damage (Becker et al., 2018; Jurado et al., 2010; McNees et al., 2005). Because RPPA showed no significant changes in DNA damage response upon BCMA loss (Fig. S2 G), we hypothesized that altered ATMIN expression may not be a result of DNA damage response but instead modulation by BCMA. To validate our Ribo-seq findings, we performed polysome fractionation to demonstrate BCMA-mediated translational changes in ATMIN expression. Ribosomes captured from MM cell lysates were separated into pooled fractions of heavy polysomes, lighter ribosomes, and monosomes to determine which fractions bound ATMIN upon the loss of BCMA expression (Fig. 2 F). Compared with the control, siBCMA-expressing MM cells showed significantly decreased ATMIN transcript binding to heavy polysomes, indicating reduced ATMIN translation efficiency (Fig. 2 G). In addition, ATMIN is exclusively regulated at the translational level by BCMA, since total ATMIN mRNA transcripts remain unchanged upon the loss of BCMA expression (Fig. 2 H).

To directly access changes in ATMIN after BCMA inhibition, we compared ATMIN protein levels in MM cells treated with siSCRM controls or two different siBCMA clones. We found strong inhibition of ATMIN protein by siBCMA compared with siSCRM (Fig. 2 I). To determine the effect of ATMIN on cell viability, we generated two independent ATMIN CRISPR knockout pools in U266 cells and observed a 30–40% reduction in cell viability. This result suggests that, like BCMA, ATMIN is also required for the viability of MM cells (Fig. 2 J).

### Wild-type soluble BCMA decoy receptor inhibits MM growth through APRIL/BCMA signaling but lacks efficacy in BAFF-driven DLBCL models

**Figure 3.**
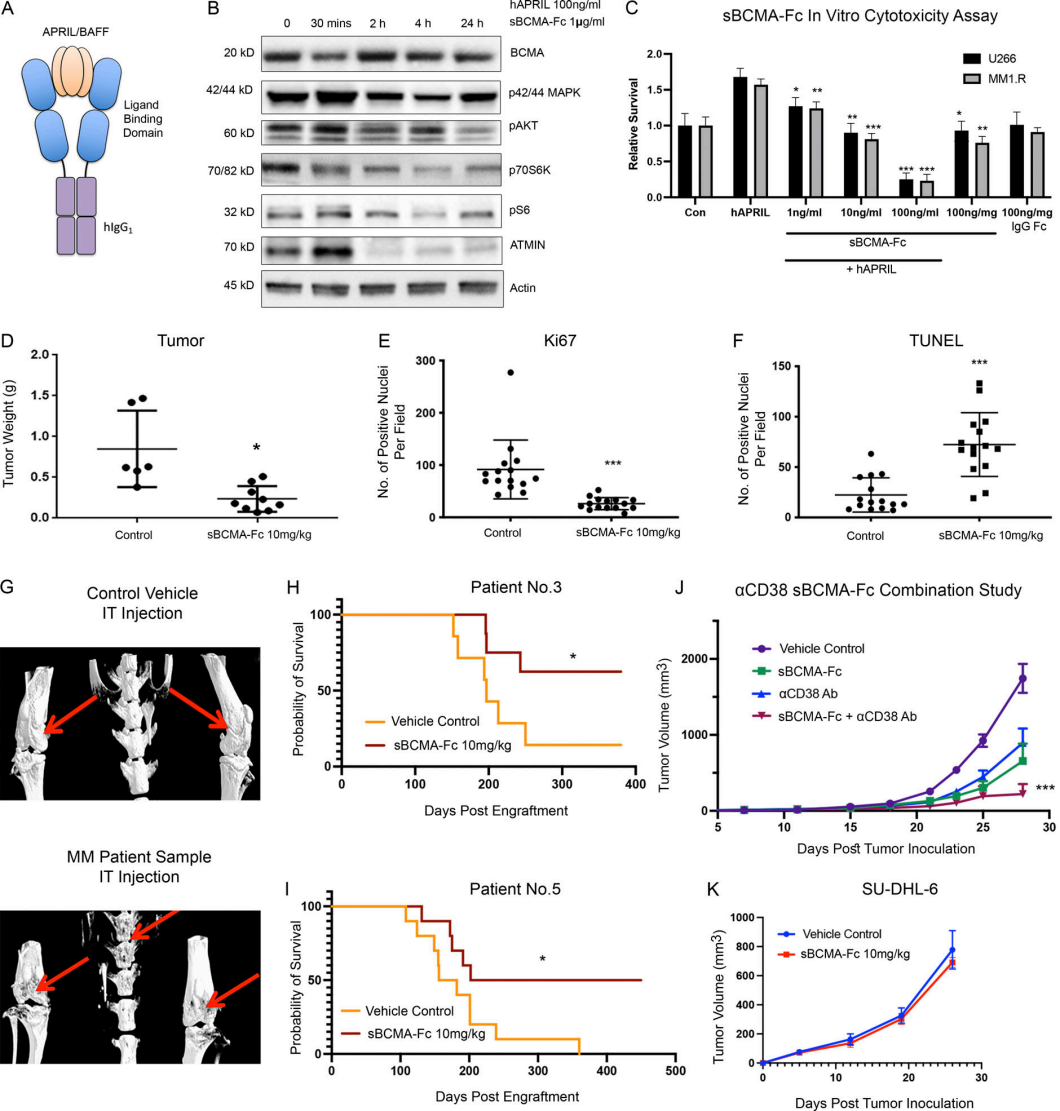
**Wild-type sBCMA decoy receptor inhibits MM growth through APRIL/BCMA signaling but lacks efficacy in BAFF-driven DLBCL model. (A)** Schematic illustrations of recombinant human sBCMA-Fc binding to human APRIL. **(B)** Analysis of BCMA downstream protein expression in U266 MM cells upon sBCMA-Fc treatment at multiple time points. **(C)** sBCMA-Fc dose-dependent cytotoxicity assay validating in vitro cell survival in the presence of increasing doses of sBCMA-Fc (P = 0.0095, 1 ng/ml; P = 0.0024, 10 ng/ml; and P = 0.0001, 100 ng/ml) and hAPRIL (100 ng/ml) in U266 and MM1.R MM cells. Cells were maintained in low (3%) FCS to reduce possible growth stimulation mediated through other growth factors present in FCS. Each sample was performed in triplicate. **(D)** Terminal tumor weight of mice inoculated with MM1.R MM tumors and treated with vehicle control or 10 mg/kg of sBCMA-Fc; P = 0.0217. **(E)** Quantification of Ki67 staining in MM1.R MM tumors and treated with vehicle control or 10 mg/kg of sBCMA-Fc; P = 0.0001. **(F)** Quantification of TUNEL staining in MM1.R MM tumors and treated with vehicle control or 10 mg/kg of sBCMA-Fc; P = 0.0001. **(G)** Representative CT scans of mice tibias, femurs, and vertebrae inoculated with control (top) or MM PDX tumor cells (bottom). Osteolytic bone degradation was observed in MM PDX injected animal (bottom image) but not in the control injected animals (top image). **(H)** Kaplan–Meier survival analysis of animals engrafted with MM cells from patient 3 showing prolonged overall survival in the sBCMA-Fc–treated group (*n* = 8) compared with the vehicle control (*n* = 7); P = 0.027. **(I)** Kaplan–Meier survival analysis of animals engrafted with MM cells from patient 5 showing prolonged overall survival in the sBCMA-Fc–treated group (*n* = 10) compared with vehicle control (*n* = 10); P = 0.0362. **(J)** Subcutaneous tumor growth of MM1.R MM tumors in 6-wk-old female NSG mice dosed with sBCMA-Fc 10 mg/kg every 48 h (*n* = 7); P = 0.0195. αCD38 10 mg/kg weekly (*n* = 7; P = 0.0238) and sBCMA-Fc and αCD38 combination (*n* = 8; P < 0.001) compared with vehicle control (*n* = 8). **(K)** Subcutaneous tumor growth of SU-DHL-6 DLBCL tumors in mice dosed with vehicle control or sBCMA-Fc 10 mg/kg every 48 h (*n* = 5). Statistical analysis was conducted using *t* test and one-way ANOVA for comparing between treatment groups. Repeated ANOVA used for changes in tumor growth over time. *, P < 0.05; **, P < 0.01; ***, P < 0.001. Source data are available for this figure: SourceData F3.

Because gain-of-function mutations in BCMA are rarely reported, BCMA signaling activity is almost exclusively regulated by its ligands APRIL and BAFF. Therefore, we investigated a ligand blocking approach, using a soluble decoy receptor comprising the BCMA extracellular domain (sBCMA) fused to the human IgG1 Fc domain (sBCMA-Fc) to trap and neutralize APRIL and BAFF (Fig. 3 A).

Treatment with sBCMA-Fc decreased downstream protein expression of similar canonical signaling regulated by the BCMA that we found through genetic inhibition of BCMA (Fig. 3 B). To determine the inhibitory effect of sBCMA-Fc on the viability of MM cells, we performed in vitro cytotoxicity assays using U266 and MM1.R cells. A concentration-dependent decrease in MM cell viability was observed when cells were treated with sBCMA-Fc and cultured in exogenous APRIL under reduced serum conditions, supporting the importance of APRIL as a growth stimulus in MM cells (Fig. 3 C). Thus, sBCMA-Fc effectively neutralized ligand-mediated activation of BCMA signaling pathways in vitro, resulting in decreased MM cell growth.

**Figure S3.**
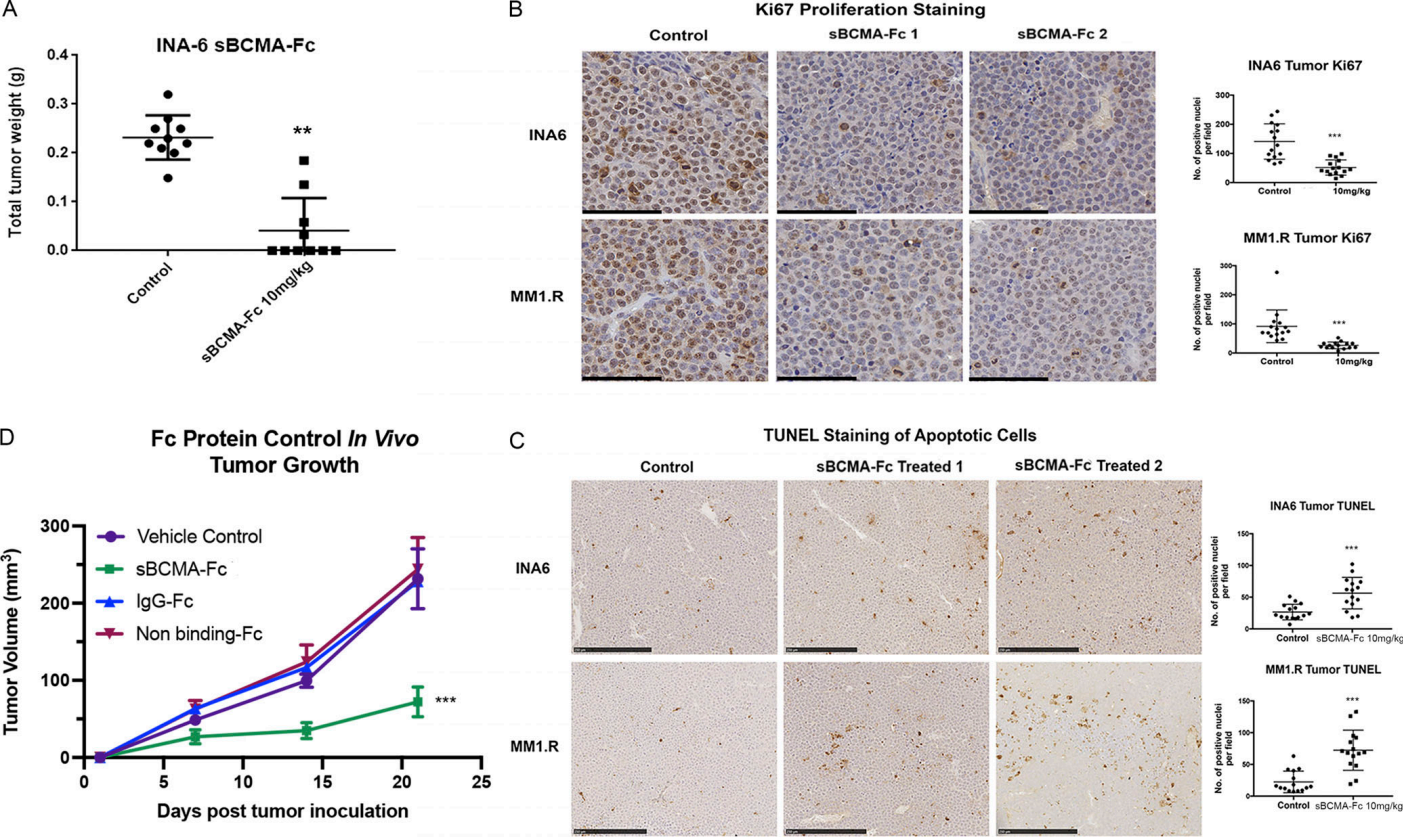
**Wild-type sBCMA decoy receptor treatment inhibits proliferation and promotes tumor cell death in MM. (A)** Terminal tumor weight of mice inoculated with INA-6 MM tumors and treated with vehicle control or 10 mg/kg of sBCMA-Fc (*n* = 10); P = 0.0082. **(B)** Representative images of Ki67-positive cells in the vehicle control and sBCMA-Fc–treated INA-6 (top panels) and MM1.R (bottom panels) MM tumors analyzed by IHC staining. Scale bar, 50 μm. Quantification of Ki67 staining on right. INA-6, P < 0.0001; MM1.R, P < 0.0001. **(C)** Representative images of TUNEL-positive cells in the vehicle control and sBCMA-Fc–treated INA-6 (top panels) and MM1.R (bottom panels) MM tumors analyzed by IHC staining. Quantification of TUNEL staining on right. INA-6, P = 0.0001; MM1.R, P < 0.0001. Scale bar, 50 μm. **(D)** Tumor growth kinetics of MM1.R tumors treated with vehicle control, sBCMA-Fc V3 (10 mg/kg; P < 0.0001), IgG-Fc control (10 mg/kg), and nonbinding Fc control (10 mg/kg; *n* = 5). Statistical analysis was conducted using *t* test and one-way ANOVA for comparing between treatment groups. Repeated ANOVA used for changes in tumor growth over time. *, P < 0.05; **, P < 0.01; ***, P < 0.001.

**Figure S4.**
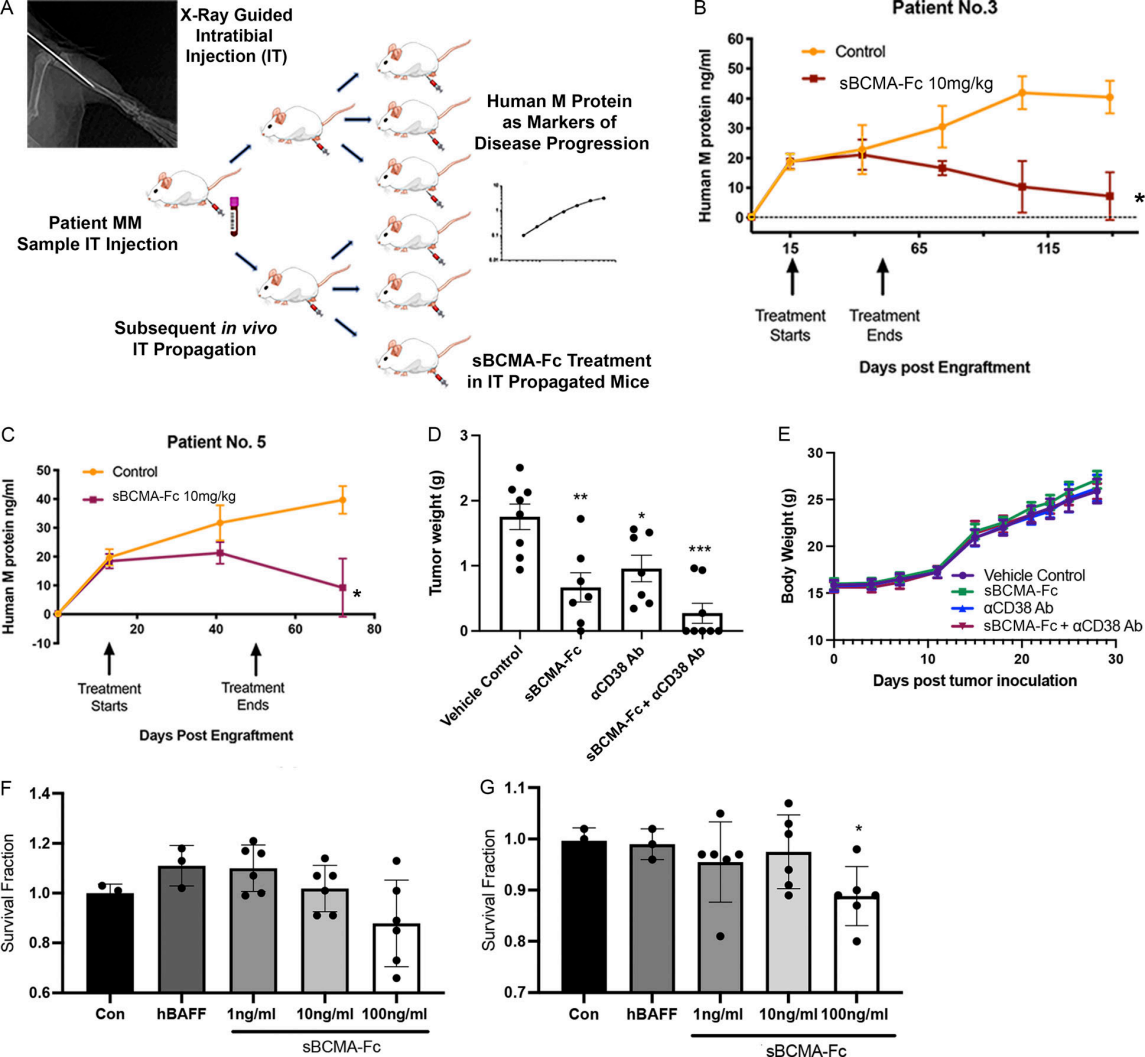
**Wild-type sBCMA decoy receptor inhibits MM growth in vivo but lacks efficacy in BAFF-driven DLBCL. (A)** Schematic flowchart of in vivo MM PDX propagation. Patient tumor cells were isolated from patient bone marrow biopsies and injected into the tibias of NSG mice. PDX were subsequently propagated in vivo using the same intratibial inoculation procedure and treated with vehicle control or sBCMA-Fc. Human IgG protein in mouse serum was continuously monitored over time as a marker of tumor progression. **(B)** Human IgG protein in mouse serum detected in animals successfully engrafted with MM cells from patient 3, showing reduction in human M protein level after sBCMA-Fc treatment (*n* = 8) compared with vehicle control (*n* = 7); P = 0.012. **(C)** Human IgG protein in mouse serum detected in animals successfully engrafted with MM cells from patient 5, showing reduction in human IgG protein level after sBCMA-Fc treatment (*n* = 10) compared with vehicle control (*n* = 10); P = 0.026. **(D)** Terminal tumor weight of MM1.R MM cells in mice dosed with sBCMA-Fc 10 mg/kg every 48 h (*n* = 7; P = 0.0078), αCD38 10 mg/kg weekly (*n* = 7; P = 0.01), sBCMA-Fc and αCD38 combination (*n* = 8; P < 0.0001), and vehicle control (*n* = 8) in 6-wk-old female NSG mice. **(E)** Changes in body weight of animals from study described in D. **(F)** sBCMA-Fc dose-dependent cytotoxicity assay validating in vitro cell survival in the presence of increasing doses of sBCMA-Fc and hBAFF (100 ng/ml) in SU-DHL-6 DLBCL cells. **(G)** sBCMA-Fc dose-dependent cytotoxicity assay validating in vitro cell survival in the presence of increasing doses of sBCMA-Fc and hBAFF (100 ng/ml) in Daudi DLBCL cells. Treatment with 100 ng/ml sBCMA-Fc led to significant reduction in cell number; P = 0.045. Statistical analysis was conducted using *t* test and one-way ANOVA for comparing between treatment groups. Repeated ANOVA used for changes in tumor growth over time. *, P < 0.05; **, P < 0.01; ***, P < 0.001.

We next examined the efficacy of sBCMA-Fc in vivo. Treatment with sBCMA-Fc resulted in a significant tumor reduction in both MM1.R (Fig. 3 D) and INA-6 MM (Fig. S3 A) tumors and was associated with decreased tumor cell proliferation (Figs. 3 E and S3 B) and increased apoptosis (Figs. 3 F and S3 C). A separate study was conducted using IgG Fc and a decoy receptor without binding to APRIL and BAFF to show the therapeutic specificity of the sBCMA-Fc molecule (Fig. S3 D). Collectively, these findings provide supporting evidence that sBCMA-Fc is efficacious as a monotherapy in xenograft models of MM. To further validate the therapeutic potential of sBCMA-Fc in human MM specimens, we established patient-derived xenografts (PDX) by engrafting freshly isolated human MM cells into the tibia of an immunodeficient murine host (Fig. S4 A). We screened eight male and three female patients, of whom six were untreated and five were treated; most had IgGκ or IgGλ myeloma type (Table S1). MM cells from patients 3 and 5 (both IgGκ myeloma type) were successfully engrafted and propagated for in vivo tumor study. After inoculation of PDX-derived MM cells, we confirmed engraftment upon the presence of human M protein (IgGκ) and proceeded with sBCMA-Fc treatment (Fig. S4, B and C). Computed tomography (CT) scans of animals with successful engraftment showed macroscopic osteolytic lesions consistent with osteopenia found in MM patients (Fig. 3 G). Engrafted mice were treated with sBCMA-Fc at 10 mg/kg every 48 h for 28 d, and tumor growth was monitored with systemic level of human IgGκ in blood serum until mice reached an ethical endpoint. In both PDX lines that were successfully propagated, treatment with sBCMA-Fc led to significant reduction in tumor expansion, decreased IgGκ signal, and prolonged overall survival, which demonstrates the ligand dependence of BCMA signaling in human MM (Fig. 3, H and I; and Fig. S4, B and C). We also evaluated the therapeutic relevance of sBCMA-Fc in combination with other MM-targeted therapies such as αCD38 antibodies. The combination of sBCMA-Fc with a CD38 therapeutic antibody (αCD38) showed superior antitumor activity compared with either monotherapy alone (Figs. 3 J and S4 D). There were no differences in body weight among the treatment groups (Fig. S4 D).

Based on published studies, BAFF is also capable of binding to BCMA receptor at a lower affinity (Bossen and Schneider, 2006). Therefore, we investigated the therapeutic efficacy of sBCMA-Fc in B cell malignancy models such as DLBCL, which is BAFF dependent (Fu et al., 2021; Lyu et al., 2010). BAFF-sensitive SU-DHL-6 and Daudi DLBCL cells were treated with ascending concentrations of sBCMA-Fc to assess in vitro cytotoxicity. Interestingly, compared with MM, DLBCL cells were less sensitive toward sBCMA-Fc treatment (Fig. S4, F and G). To determine the efficacy of sBCMA-Fc in DLBCL tumor models, mice bearing subcutaneous SU-DHL-6 tumors were treated with sBCMA-Fc at 10 mg/kg every 48 h, which is the efficacious dose in MM tumors. However, treatment with sBCMA-Fc at 10 mg/kg did not provide significant antitumor benefit in BAFF-sensitive SU-DHL-6 lymphomas (Fig. 3 K). Knowing that MM is primarily APRIL driven, while DLBCL is BAFF dependent, we hypothesized that the sBCMA-Fc molecule lacks sufficient binding affinity toward BAFF and hence failed to inhibit BAFF-mediated signaling.

### Engineering a high-affinity decoy receptor fusion protein against APRIL and BAFF

**Figure 4.**
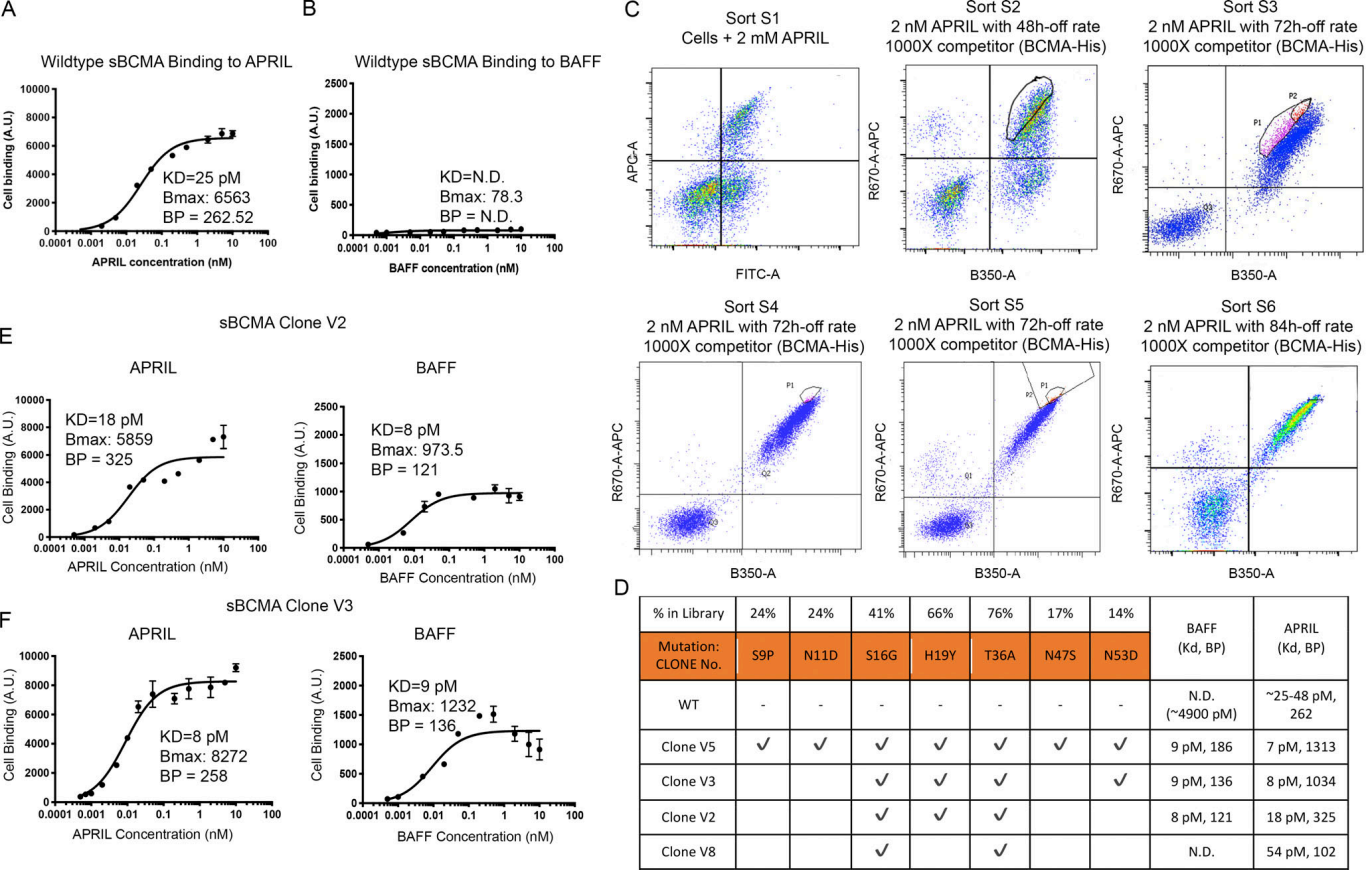
**Engineering high-affinity decoy receptor fusion protein against APRIL and BAFF. (A)** Flow cytometry–based binding curve showing yeast-displayed wild-type sBCMA binding to increasing concentrations of APRIL. Calculated *K*_*D*_, *B*_max_, and binding potential (BP) is also shown. A.U., arbitrary units. **(B)** Flow cytometry–based binding curve showing yeast-displayed wild-type sBCMA binding to increasing concentrations of BAFF. Calculated *K*_*D*_, *B*_max_, and BP is shown. **(C)** Overlaid flow cytometry dot plots representing sorting strategies of yeast-displayed sBCMA library binding to 2 nM APRIL in six consecutive sorts with 48–84 h off-rate binding and 1,000× competitor. Gated populations are collected from each sort and propagated for the next sorting round. **(D)** Binding affinities to APRIL and BAFF of wild-type sBCMA and selected mutant sBCMA clones. Conserved amino acid mutations were identified in mutant clones, and the frequency of occurring mutations is also listed. **(E)** Binding curve of high-affinity mutant sBCMA clone V2 binding to increasing concentrations of APRIL (left) and BAFF (right). Calculated *K*_*D*_, *B*_max_, and BP is shown. **(F)** Binding curve of high-affinity mutant sBCMA clone V3 binding to increasing concentration of APRIL (left) and BAFF (right). Calculated *K*_*D*_, *B*_max_, and BP is shown.

To validate whether the lack of antitumor activity demonstrated by sBCMA-Fc against DLBCL is due to weak binding affinity toward BAFF, we evaluated the binding kinetics of sBCMA to hAPRIL and hBAFF. While the *K*_*D*_ between sBCMA and hAPRIL has been reported to be 25–48 pM (Hymowitz et al., 2005; Yu et al., 2000), the *K*_*D*_ between sBCMA and hBAFF was weak and was experimentally estimated to be ∼4,900 pM (Fig. 4, A and B). This finding supported our hypothesis that weak binding between sBCMA-Fc and BAFF is likely to be a contributing factor toward poor therapeutic efficacy in BAFF-driven DLBCL models.

**Figure S5.**
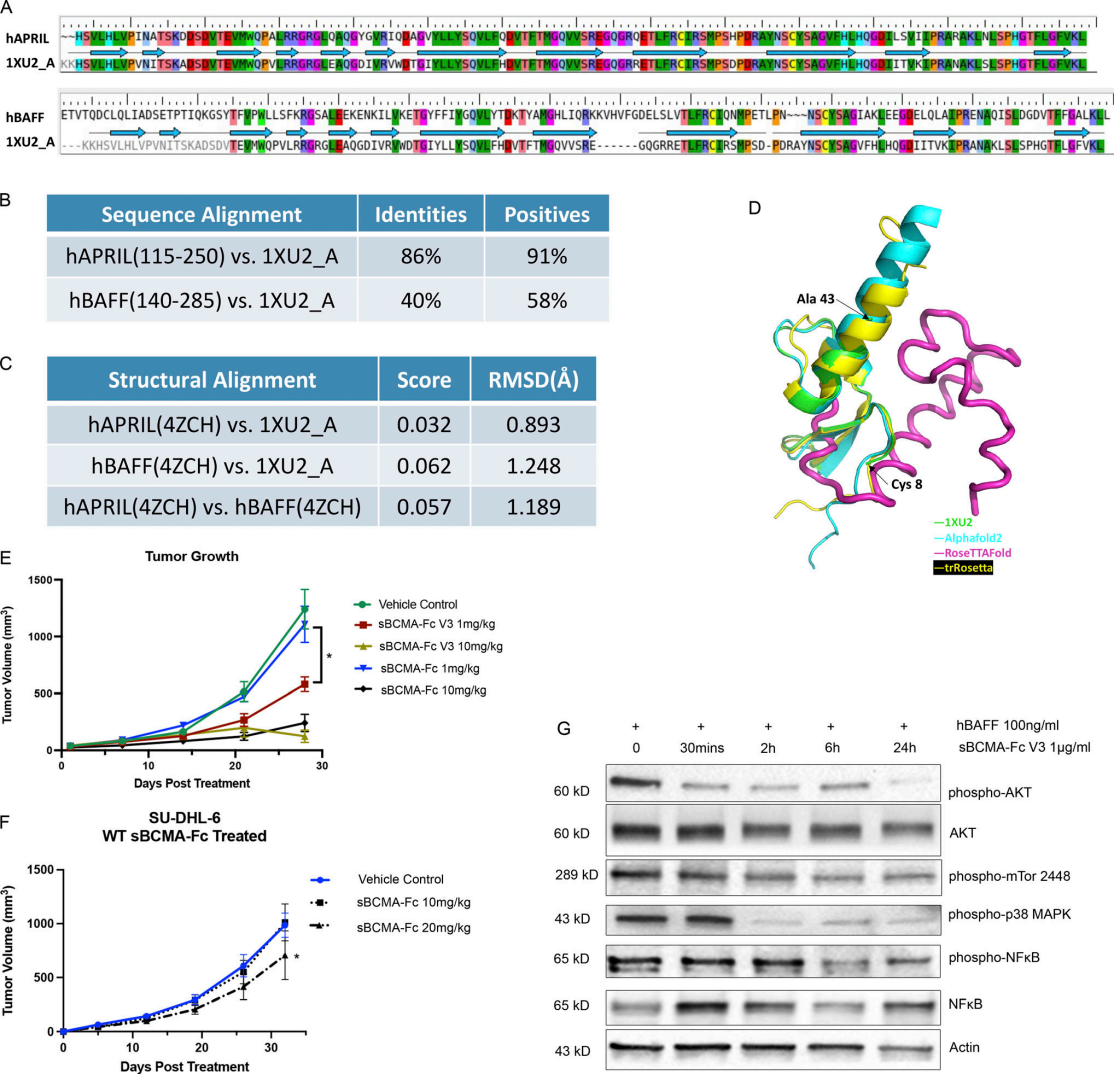
**Affinity-enhanced sBCMA-Fc V3 treatment inhibits tumor growth in models of MM and DLBCL. (A)** Amino acid sequence alignment between hAPRIL (top) or hBAFF (bottom) and Protein Data Bank structural ID 1XU2 (structure of mAPRIL and human sBCMA cocomplex). PDB 4ZCH reported a single-chain human APRIL-BAFF-BAFF heterotrimer structure. **(B)** Quantification of sequence alignment between hAPRIL/hBAFF and published structure 1XU2. **(C)** Quantification of structural alignment between hAPRIL/hBAFF and published structure 1XU2 as well as between predicted hAPRIL and hBAFF structures. **(D)** Structure overlay of extracellular BCMA flexible region (aa 44–54) between 1XU2 and structures predicted using Alphafold2 (cyan), RoseTTAfold (magenta), and trRosetta (yellow). **(E)** Subcutaneous tumor growth of MM1.R MM tumors in 6-wk-old female NSG mice dosed with wild-type sBCMA-Fc at 1 and 10 mg/kg every 48 h (*n* = 5), sBCMA-Fc V3 at 1 and 10 mg/kg every 48 h (*n* = 5), and vehicle control (*n* = 5). sBCMA-Fc V3 treatment significantly reduced tumor growth at 1 mg/kg; P = 0.031. **(F)** Subcutaneous tumor growth of SU-DHL-6 DLBCL in mice dosed with vehicle control, sBCMA-Fc 10 mg/kg, and sBCMA-Fc 20 mg/kg every 48 h (*n* = 5). sBCMA-Fc treatment significantly reduced tumor growth at 20 mg/kg; P = 0.043. **(G)** Analysis of BCMA downstream protein expression in SU-DHL-6 DLBCL cells upon sBCMA-Fc V3 treatment at multiple time points. Statistical analysis was conducted using *t* test and one-way ANOVA for comparing between treatment groups. Repeated ANOVA used for changes in tumor growth over time. *, P < 0.05; **, P < 0.01; ***, P < 0.001. Source data are available for this figure: SourceData FS5.

To address this shortcoming, we engineered a sBCMA mutant with the capability of neutralizing both APRIL and BAFF at high affinity. Using low-fidelity Taq polymerase-based error-prone PCR as previously described (Miao et al., 2021), nucleotide mutations were randomly generated into the extracellular domain of BCMA gene from amino acid 1 (methionine) to 54 (alanine). The resulting library displayed on the yeast surface was analyzed by FACS to isolate clones with desired binding characteristics. We carried out initial affinity screening using hAPRIL, because it was previously reported that the BCMA binding site to APRIL and BAFF shares high homology (Fig. S5 A). Therefore, we hypothesized that clones identified in this screening process will likely have high binding affinity to both APRIL and BAFF (Liu et al., 2003). Six rounds of FACS sorting were performed sequentially, and 1–3% clones with the highest binding to APRIL were enriched from each sort and propagated for the subsequent sorting round (Fig. 4 C).

The top 118 clones selected from sort rounds 3–6 were sequenced and analyzed for consensus mutations (Tables S2, S3, S4, and S5). Overall, seven consensus mutations were identified (Fig. 4 D). As hypothesized, we observed a dramatic improvement in the binding affinities of candidate mutant clones toward both APRIL and BAFF. Ultimately, the mutant sBCMA V3 carrying mutations S16G, H19Y, T36A, and N53D was selected as our top candidate because it possessed fewer mutations while still retaining high binding affinity toward APRIL and BAFF. The sBCMA V2 without N53D mutation resulted in a slightly weaker binding to APRIL (Fig. 3, E and F).

### Structural and biological characterization of affinity-enhanced sBCMA-Fc V3

**Figure 5.**
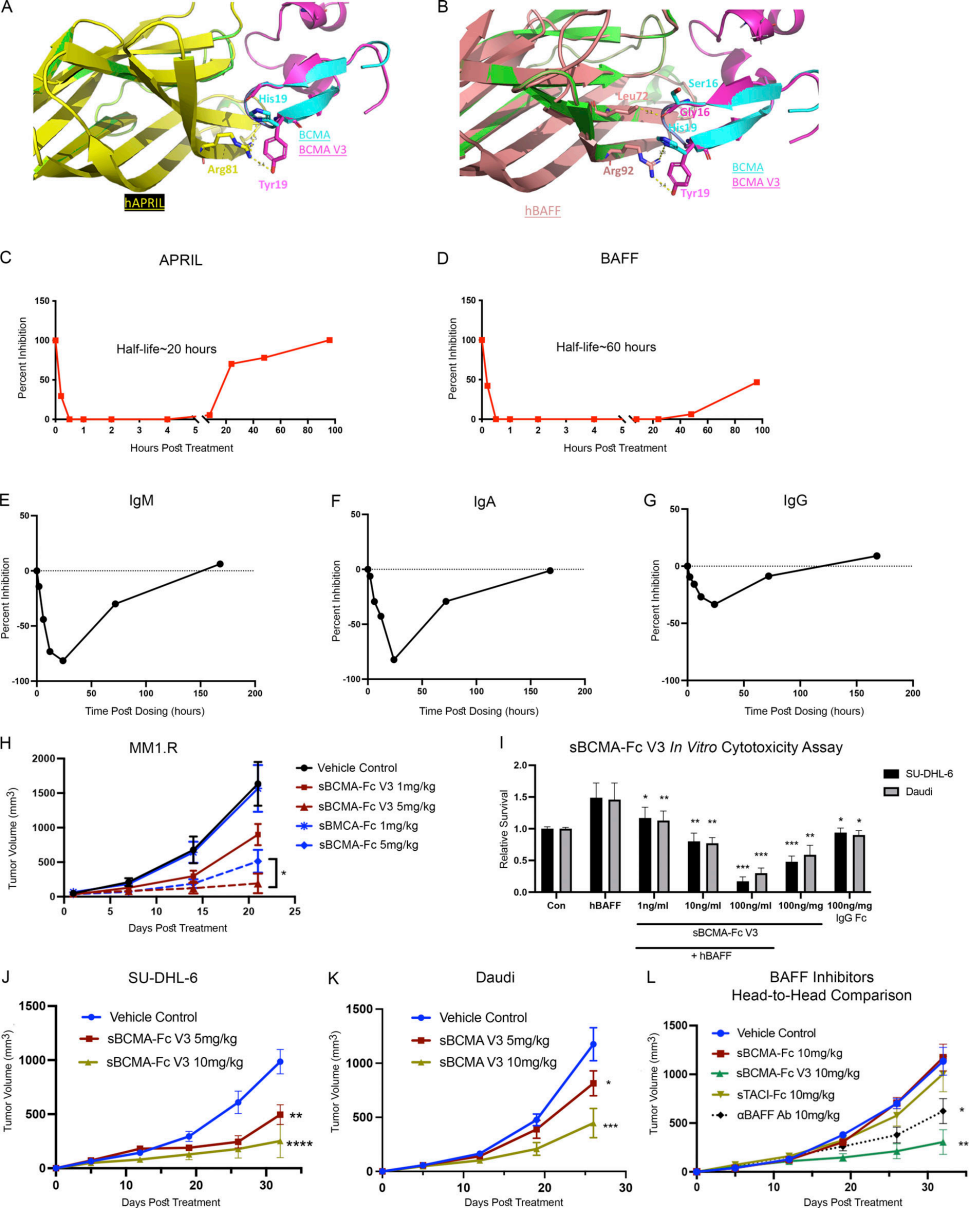
**Structural and biological characterization of affinity-enhanced sBCMA-Fc V3. (A)** Computational modeling of sBCMA V3 mutant clone (magenta) in cocomplex with hAPRIL (yellow) overlaying on PDB structure 1XU2 consisting of hBCMA (cyan) in cocomplex with mAPRIL (green). Predicted binding interaction between hAPRIL (yellow) and sBCMA V3 (cyan) showing Arg81 on hAPRIL interacting with H19Y on sBCMA V3. **(B)** Computational modeling of sBCMA V3 mutant clone (magenta) in cocomplex with hBAFF (salmon) overlaying on PDB structure 1XU2 consisting of human BCMA (cyan) in cocomplex with mAPRIL (green). Predicted binding interaction between hBAFF (salmon) and sBCMA V3 (cyan) showing Arg93 on hBAFF interacting with H19Y on sBCMA V3. **(C)** Serum level of APRIL in mouse serum after a single dose of sBCMA-Fc V3 at 10 mg/kg. Each data point represents duplicate repeats collected at each time point. **(D)** Serum level of BAFF in mouse serum after a single dose of sBCMA-Fc V3 at 10 mg/kg. Each data point represents duplicated repeats collected at each time point. **(E)** Mouse serum IgM levels after sBCMA-Fc V3 treatment. **(F)** Mouse serum IgA levels after sBCMA-Fc V3 treatment. **(G)** Mouse serum IgA levels after sBCMA-Fc V3 treatment. **(H)** Subcutaneous tumor growth of MM1.R MM tumors in 6-wk-old female NSG mice dosed with wild-type sBCMA-Fc at 1 and 5 mg/kg every 48 h (*n* = 5), sBCMA-Fc V3 at 1 and 5 mg/kg every 48 h (*n* = 5), and vehicle control (*n* = 5). MM1.R tumors treated with sBCMA-Fc V3 showed significant reduction in tumor growth compared with sBCMA-Fc treatment at the same concentration; P = 0.0365. **(I)** sBCMA-Fc V3 dose-dependent cytotoxicity assay validating the in vitro cell survival in the presence of increasing doses of sBCMA-Fc V3 and hBAFF (100 ng/ml) in SU-DHL-6 and Daudi DLBCL cells (P = 0.032; 1 ng/ml; P = 0.075, 10 ng/ml; P = 0.0001, 100 ng/ml). Cells were maintained in low (3%) FCS to reduce possible growth stimulation mediated through other growth factors present in FCS. Each sample was performed in triplicate. **(J)** Subcutaneous tumor growth of SU-DHL-6 DLBCL tumors in 6-wk-old female *NOD-scid* mice dosed with 5 or 10 mg/kg sBCMA-Fc V3 every 48 h (*n* = 5) and vehicle control (*n* = 5), P < 0.0001. **(K)** Subcutaneous tumor growth of Daudi DLBCL tumors in 6-wk-old female *NOD-scid* mice dosed with 5 or 10 mg/kg sBCMA-Fc V3 every 48 h (*n* = 5) and vehicle control (*n* = 5); P = 0.0001. **(L)** In vivo head-to-head comparison of antitumor efficacies in subcutaneous SU-DHL-6 DLBCL tumors treated with 10 mg/kg wild-type sBCMA-Fc every 48 h (*n* = 5), 10 mg/kg sBCMA-Fc V3 every 48 h (*n* = 5; P < 0.0001), 10 mg/kg sTACI-Fc every 48 h (*n* = 5), and 10 mg/kg αBAFF antibody twice a week (*n* = 5). Subcutaneous tumor growth was monitored throughout the study. Statistical analysis was conducted using *t* test and one-way ANOVA for comparing between treatment groups. Repeated ANOVA used for changes in tumor growth over time. *, P < 0.05; **, P < 0.01; ***, P < 0.001.

To characterize the structural, biophysical, and biological properties of our affinity-enhanced clone sBCMA V3, we performed computational model simulations of sBCMA-Fc V3 in cocomplex with APRIL and BAFF. The sequence alignment and subsequent modeling based on the wild-type hBCMA and mAPRIL complex structures were obtained from PDB 1XU2; Fig. S5 A; Hymowitz et al., 2005; Schuepbach-Mallepell et al., 2015). Structural alignment between hAPRIL and mAPRIL revealed high structural homology with a root mean square deviation (RMSD) of 0.893 Å. While hBAFF and mAPRIL were not highly conserved in their primary sequence (Fig. S5 B), they shared high similarity on tertiary structure, with RMSD of 1.248 Å (Fig. S5 C). This is consistent with a previous report that hAPRIL and hBAFF share a similar binding site at the DxL motif of BCMA (Gordon et al., 2010). We found that residues 44–54 are likely to be a highly flexible. Of the four mutations we identified, mutations S16G and H19Y are located within the BCMA binding motif, and T36A and N53D are located outside of the binding motif. Mutations S16G, H19Y, and T36A both individually and collectively led to an improvement in the binding affinity, stability, and thermodynamic interaction of sBCMA V3/hAPRIL and sBCMA V3/hBAFF cocomplexes (Fig. 5 A and Table 1; Fig. 5 B and Table 2). Based on protein interaction modeling, the H19Y mutation is likely to be the most important contributor responsible for improved binding affinity to both hAPRIL and hBAFF. While residue 19 was reported as a critical residue within the sBCMA/BAFF cocomplex (Bossen and Schneider, 2006), no such analysis has been done with sBCMA/APRIL cocomplex. Furthermore, we found that amino acid substitution of H19Y on sBCMA is the critical mutation responsible for enhanced binding toward both APRIL and BAFF (Tables 1 and 2). Surface complementarity analysis between wild-type sBCMA, sBCMA V3, hAPRIL, and hBAFF was performed. We found that the H19Y mutation improved surface complementarity of sBCMA V3 toward both hAPRIL and hBAFF (Table 3). In its cocomplex form, the H19Y mutation shortened the intramolecular distance between sBCMA and hAPRIL. Similarly, the intramolecular distance between sBCMA V3 and hBAFF was also reduced, corresponding to a tighter binding cocomplex structure (Table 3).

**Table 1.** Calculated binding affinity and protein stability (in kcal/mol) for each BCMA mutation in cocomplex with APRIL

Residue	Original	Mutated	Δ Affinity	Δ Stability (solvated)	Total ΔG value (original/mutated)
R:16	SER	ALA	−0.13	−1.78	N/A
R:19	HIS	TYR	−3.95	−3.59	N/A
R:36	THR	ALA	−0.22	4.91	N/A
R:16 R:19 R:36	SER HIS THR	ALA TYR ALA	−5.85	−1.19	−4.333/−33.041

N/A, not applicable.

**Table 2.** Calculated binding affinity and protein stability (in kcal/mol) for each BCMA mutation in cocomplex with BAFF

Residue	Original	Mutated	Δ Affinity	Δ Stability (solvated)	Total ΔG value (original/mutated)
R:36	THR	ALA	−0.14	4.98	N/A
R:19	HIS	TYR	−7.73	−7.52	N/A
R:16	SER	GLY	1.38	1.03	N/A
R:16 R:19 R:36	SER HIS THR	GLY TYR ALA	−3.55	2.8	−17.462/−19.858

N/A, not applicable.

**Table 3.** Calculated surface complementarity and molecular distance between BCMA V3 and APRIL/BAFF

Site in BCMA	Surface complementarity to hAPRIL		Distance to hAPRIL		Surface complementarity to hBAFF		Distance to hBAFF	
	WT	Single mutant	WT	Single mutant	WT	Single mutant	WT	Single mutant
S16G	0.65	0.51	N/A	N/A	0.76	0.76	3.2 Å (to Leu72)	3.2 Å (to Leu72)
H19Y	0.88	0.21	3.8 Å (to Arg81)	2.1 Å (to Arg81)	0.88	0.2	3.7 Å (to Arg92)	2.0 Å (to Arg92)
T36A	0.00	0.00	N/A	N/A	N/A	N/A	N/A	N/A

N/A, not applicable.

To establish the therapeutic potency of affinity-enhanced mutant clone sBCMA V3 in models of MM and B cell malignancies, we first generated a sBCMA V3 + hIgG1 fusion protein (sBCMA-Fc V3) for improved stability and bioavailability. The pharmacokinetics of sBCMA-Fc V3 was validated in *NOD-scid* immunocompromised non–tumor-bearing mice. A single dose of 10 mg/kg sBCMA-Fc V3 injected into non–tumor-bearing animals completely suppressed the level of APRIL and BAFF within 30 min. Over time, sBCMA-Fc V3 showed an inhibitory half-life of ∼20 h with APRIL suppression (Fig. 5 C) and 60 h with BAFF inhibition (Fig. 5 D). This finding is consistent with the half-life of human decoy receptors in mice reported previously (Kariolis et al., 2017; Miao et al., 2021). Inhibition of APRIL and BAFF is known to suppress B cell–mediated immunoglobulin class switching, leading to decreased Ig levels (Castigli et al., 2004; Castigli et al., 2005; Grasset et al., 2020). Here, we examined the expression of IgM, IgA, and IgG in mouse serum after a single 10-mg/kg dose of sBCMA-Fc V3. A transient reduction of IgM, IgA, and IgG was observed within 48 h after treatment and returned to predosing level within 7 d, suggesting this treatment effect is reversible (Fig. 5, E–G).

To investigate whether sBCMA-Fc V3 can improve antitumor activity in MM models compared with wild-type sBCMA-Fc, we tested various doses of wild-type sBCMA-Fc or sBCMA-Fc V3 on MM tumor growth. A significant improvement in antitumor activity was observed in sBCMA-Fc V3–treated groups but not in wild-type treated with sBCMA-Fc at lower doses (Fig. 5 H). Interestingly, the antitumor affects were comparable when animals were treated with both molecules at a higher dose of 10 mg/kg, suggesting that both treatments reached a plateau in achieving ligand/receptor inhibition (Fig. S5 E). Because MM progression is primarily APRIL driven, sBCMA-Fc V3 with enhanced APRIL binding resulted in more efficient APRIL neutralization at lower concentration.

Next, we investigated whether sBCMA-Fc V3 with a 500-fold improvement in its binding affinity to BAFF (9 pM) would increase antitumor activity in BAFF-driven DLBCL models. sBCMA-V3 Fc–mediated inhibition of BAFF signaling pathway was evaluated in vitro in BAFF-dependent SU-DHL-6 and Daudi DLBCL cell lines. sBCMA-Fc V3 treatment effectively reduced DLBCL cell growth in a dose-dependent manner, suggesting these cells rely on BAFF for growth and survival (Fig. 5 I). Treatment with sBCMA-Fc V3 also led to decreased expression of pAKT, p38-MAPK, and pNF-κB p65, all of which are critical molecular components of the BCMA/TACI/BAFF-R canonical signaling pathway that governs malignant B cell growth (Hatzoglou et al., 2000; Fig. S5 G). To determine the therapeutic efficacy of sBCMA-V3 Fc in vivo, SU-DHL-6 and Daudi tumor models were established by subcutaneous engraftment in *NOD-scid* mice. Animals were treated with 5 or 10 mg/kg of sBCMA-Fc V3 and compared with the vehicle control. In both DLBCL tumor models, sBCMA-Fc V3 treatment resulted in significant dose-dependent reduction in tumor size (Fig. 5, J and K). To further study the therapeutic potential of wild-type sBCMA-Fc compared with sBCMA-Fc V3 in DLBCL treatment, we evaluated the growth kinetics of SU-DHL-6 tumors treated with higher doses of wild-type sBCMA-Fc, at 10 and 20 mg/kg. Similar to what we observed in previous studies (Fig. 3 K), 10 mg/kg wild-type sBCMA-Fc had little effect on tumor growth, but tumor reduction was observed in the 20 mg/kg treatment group (Fig. S5 F). These data provide further evidence that wild-type sBCMA-Fc has suboptimal BAFF neutralizing capabilities and requires significantly higher concentrations to inhibit BAFF and DLBCL tumor growth.

To further investigate the therapeutic efficacy of sBCMA-Fc V3, we compared it to other therapeutic agents known to inhibit BAFF in a head-to-head study. These other agents were a recombinant soluble TACI-Fc (sTACI-Fc) decoy receptor that binds APRIL and BAFF at 6.4 nM and 160 pM, respectively (Wu et al., 2000), as well as an antibody against BAFF (αBAFF Ab) with a *K*_*D*_ of 0.995 nM and no reported binding to APRIL (Shin et al., 2018). The clinical versions of both molecules are currently used for the treatment of various B cell–related autoimmune diseases (Bag-Ozbek and Hui-Yuen, 2021; Barratt et al., 2020; Lee and Amengual, 2020). In this study, we compared the antitumor efficacy of sBCMA-Fc V3, sTACI-Fc, αBAFF Ab, and wild-type sBCMA-Fc in SU-DHL-6 DLBCL tumors. All therapeutic groups were treated with the same dose of 10 mg/kg, three times per week for decoy receptors and two times per week for antibody. We found that sBCMA-Fc V3 treatment resulted in significant and robust tumor reduction compared with vehicle treatment. In comparison, sTACI-Fc–treated groups showed a trend toward reduced tumor growth that did not reach statistical significance. In contrast, αBAFF Ab–treated animals showed a modest and statistically significant reduction in tumor growth. Consistent with our previous observation, wild-type sBCMA-Fc did not show significant therapeutic benefit, owing to insufficient BAFF binding (Fig. 5 L).

In summary, sBCMA-Fc V3 with enhanced binding affinity to APRIL and BAFF resulted in better antitumor activity in both APRIL-driven MM and BAFF-driven DLBCL models. In particular, compared with wild-type sBCMA-Fc, sTACI-Fc, and αBAFF Ab, sBCMA-Fc V3 treatment resulted in superior antitumor efficacy in a DLBCL model, likely driven by its strong binding toward BAFF. This observation further supports the therapeutic potential of sBCMA-Fc V3 as a treatment for MM and DLBCL.

### sBCMA-Fc V3 demonstrates adequate toxicity profile and on-target mechanism of action in nonhuman primates

Nonhuman primate toxicity studies are particularly useful because primates often respond in a physiologic manner similar to humans. To evaluate the translational potential of sBCMA-Fc V3, we conducted a single-dose toxicity study in cynomolgus monkeys to investigate drug-mediated acute toxicity after i.v. infusion of sBCMA-Fc V3.

**Figure 6.**
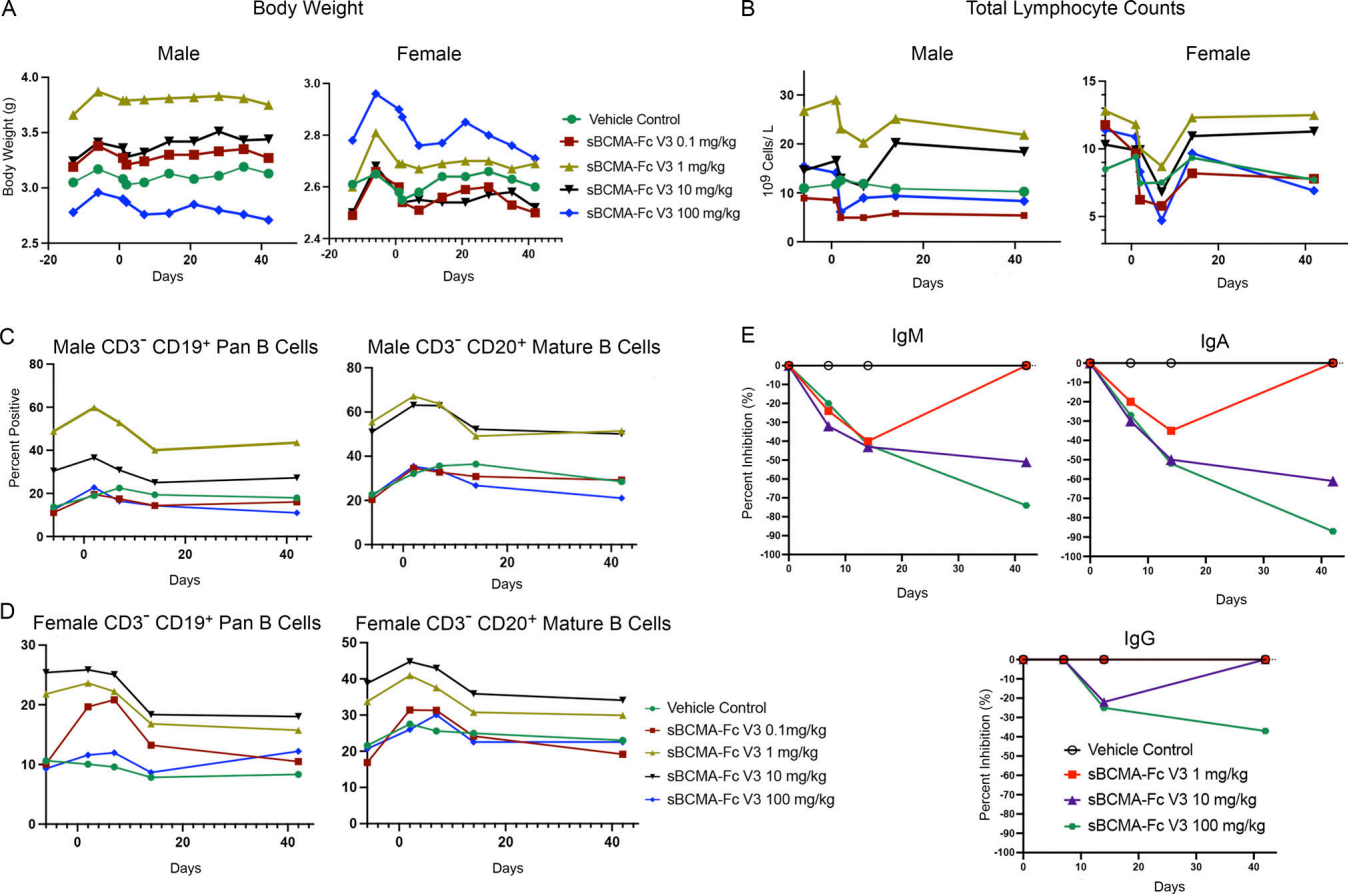
**sBCMA-Fc V3 demonstrates adequate toxicity profile and on-target mechanism of action in nonhuman primates. (A)** Changes in total body weight in kilograms of male (left) and female (right) cynomolgus monkey test subjects throughout the experiment and observation period. **(B)** Total lymphocyte counts expressed as 10^9^ cells/liter in the blood of male (left) and female (right) cynomolgus monkey test subjects. **(C)** Counts of CD3^−^CD19^+^ pan B lymphocytes (left) and CD3^−^CD20^+^ mature B lymphocytes in the peripheral blood of male cynomolgus monkey test subjects throughout the experimental period. **(D)** Counts of CD3^−^CD19^+^ pan B lymphocytes (left) and CD3^−^CD20^+^ mature B lymphocytes in the peripheral blood of male cynomolgus monkey test subjects throughout the experimental period. **(E)** Levels of IgM, IgA, and IgG levels in female cynomolgus monkey throughout the experimental period. Statistical analysis not applicable.

One animal from each sex was assigned in each of five groups and given a single i.v. infusion of sBCMA-Fc V3 (0.1, 1, 10, and 100 mg/kg) or vehicle (Table 4). Animals were observed for 2 wk before and 6 wk after treatment. Parameters evaluated include body weight, food consumption, hematology, lymphocyte immunophenotype, immunoglobulin production, and gross pathology. Blood samples were collected from each animal on days −13, −6, −3, and 1 before dosing and days 2, 7, 14, and 42 during dosing. Overall, there was no unscheduled deaths in the study, and no drug-related abnormalities of body weight or food consumption were observed for the animals in any group during the observation period (Fig. 6 A). Hematology analysis showed modest declines of RBC, hemoglobin (HGB), and hematocrit (HCT) in female and male monkeys of each group on days 2, 7, and/or 14 (Tables S6, S7, S8, and S9). However, after factoring in the total volume of blood sampled during the experiment, it was likely that the decreases in RBC, HGB, and HCT were related to blood sampling. No other hematological abnormalities were observed. Upon lymphocyte analysis, we observed a dose-dependent, transient reduction in total lymphocyte numbers in female monkeys on day 7 after dose followed by a full recovery by day 14. A similar but less significant trend was observed in male monkeys (Fig. 6 B). We further investigated B lymphocyte subpopulations, including CD19^+^ pan B cells and CD20^+^ mature B cells in male (Fig. 6 C) and female (Fig. 6 D) monkeys, with no significant changes observed in any treatment group. No gross abnormal tissue pathology was present in animal tissues examined during necropsy.

**Table 4.** sBCMA-Fc V3 single-dose toxicology study design

Group	Number of animals		Animal number		Treatment	Dose level (mg/kg)	Concentration (mg/ml)	Dose volume (ml/kg)
	Male	Female	Male	Female				
1	1	1	1,101	2,102	Vehicle	0	0	10
2	1	1	1,203	2,204	sBCMA-Fc V3	0.1	0.01	10
3	1	1	1,305	2,306	sBCMA-Fc V3	1	0.1	10
4	1	1	1,407	2,408	sBCMA-Fc V3	10	1	10
5	1	1	1,509	2,510	sBCMA-Fc V3	100	10	10

Body weight recording: predose days −14, −6; after-dose days 1, 2, 7, 14, 21, 28, 35, and 42. Blood sample collection: predose days −13, −16, 1; after-dose days 2, 7, 14, and 42.

A dose-dependent reduction in immunoglobulin pharmacodynamic markers was established in nonhuman primates treated with sBCMA-Fc V3, which led to a reduction in IgA in both male and female monkeys over time. To a lesser extent, a similar result was observed with IgM and IgG (Fig. 6 E). Consistent with our previous observation in mice (Fig. 5, E–G), these observations confirmed the on-target mechanism of sBCMA-Fc V3 on APRIL- and BAFF-mediated immunoglobulin production and class switching, confirming that the inhibition of APRIL/BAFF-mediated signaling can decreased immunoglobulin production in MM (Fig. 1 J). This finding supports our hypothesis that sBCMA-Fc V3 is a potent APRIL and BAFF inhibitor for the treatment of B cell–driven diseases in oncology and beyond.

## Discussion

The aberrant activation of APRIL and BAFF leads to dysregulated B cell growth; therefore, they are attractive therapeutic targets for B cell malignancies and autoimmune diseases (Bolkun et al., 2014; Briones et al., 2002; Carpenter et al., 2013; Chiu et al., 2007). However, the multifaceted ligand/receptor interactions between APRIL and BAFF and their receptors BCMA, BAFF-R, and TACI are particularly challenging to overcome. Past strategies have used ligand neutralization approaches with monoclonal antibodies or decoy receptors for BCMA, BAFF-R, and TACI in a limited number of disease applications (Rossi et al., 2009; Shrestha et al., 2021; Tai and Anderson, 2019). We hypothesized that a soluble BCMA decoy receptor with endogenous, strong affinity to APRIL can successfully target APRIL-driven MM tumors despite the heterogenous cytogenetic profile of MM. However, we found that this same sBCMA-Fc is ineffective against BAFF-driven DLBCL because of its weak binding to BAFF. To overcome the affinity barrier, we used a yeast surface display-based protein engineering approach and generated mutant sBCMA clones with ultra-high binding affinity against both APRIL and BAFF. The resultant therapeutic candidate sBCMA-Fc V3 showed superior antitumor activities for both APRIL-driven MM and BAFF-driven DLBCL, with a desirable safety profile.

The biological dependence of BCMA signaling on MM cell is known, but it is more commonly used as a pan-MM surface marker for targeted delivery of cytotoxic therapeutics (Tai and Anderson, 2019). Here, we provide new insight into the global translational landscape of MM cells upon the loss of BCMA and identify a distinct subgroup of proteins that are translationally controlled, which otherwise would not be detected by measuring changes in total mRNA transcripts (Cenik et al., 2015; Ingolia et al., 2019). Specifically, ATMIN (ATM-interacting protein, ASCIZ) was one of the proteins translationally regulated by BCMA. While ATMIN plays an important role in the DNA damage response (McNees et al., 2005), in B cells, ATMIN can interact with dynein light chain subunit (Dynll1) in an ATM-independent manner (Jurado et al., 2012a; Jurado et al., 2012b). In addition, recent reports indicate that ATMIN and Dynll1 are involved in the development of B cell lymphoma, providing a link between dysregulated B cell development, BCMA, and ATMIN signaling in MM (Jurado et al., 2012b). While our ribosome profiling has identified a subset of cellular proteins regulated by BCMA signaling, we have identified ATMIN as a new target of BCMA that is regulated in a translation-specific manner and is important for MM viability. However, while it is beyond the scope of the study, the causal relationship between BCMA knockdown, altered cell proliferation. and impaired protein translation remain to be fully elucidated and warrants further investigation.

To improve sBCMA binding toward BAFF, we generated a high-affinity mutant sBCMA V3 with low picomolar binding toward both APRIL and BAFF. Computational structural analysis revealed that H19Y on sBCMA V3 contributed toward enhanced binding to both ligands. Although H19 was previously reported as an important residue for binding between BCMA and BAFF, its interaction with APRIL was not described (Bossen and Schneider, 2006). Furthermore, the H19Y mutation was selected from APRIL-driven affinity maturation, indicating that H19Y on BCMA is a highly desirable mutation for achieving stronger binding to both APRIL and BAFF. This finding provides the impetus to solve the crystal structures of sBCMA V3 in cocomplex with APRIL and BAFF, which may provide additional biophysical evidence to explain how these mutations provide affinity enhancement. Therapeutically, enhancement in BAFF binding and its associated structural modifications shifted the treatment paradigm of sBCMA-Fc from a treatment selective for MM to a therapeutic candidate suited for targeting multiple B cell–driven diseases. The biological function of BAFF and APRIL is not limited to B cell malignancy but extends to autoimmune disorders and other diseases triggered by pathological B cells, suggesting a much broader clinical indication for sBCMA-Fc V3 (Samy et al., 2017).

Treatment with sBCMA-Fc V3 was found to be well tolerated in a single-dose toxicity study conducted in cynomolgus monkeys. More importantly, a dose-dependent reduction in immunoglobulin production was observed, likely due to inhibition of APRIL- and BAFF-driven Ig class switching. Furthermore, a greater depletion of IgA was observed in animals treated with sBCMA-Fc V3. Considering that a previous study showed the impact of BAFF and APRIL on mouse IgA response in the gut (Grasset et al., 2020), which is the primary site of IgA production, the modulatory effect of sBCMA-Fc V3 on gut-associated IgA production warrants further investigation. Overall, these data provide an explanation for the inhibition of BCMA signaling leading to reduced abnormal immunoglobulin production, a key feature of MM pathogenesis. In summary, sBCMA-Fc V3 was found to be safe and well tolerated in all treated animals for ≤42 d, with no significant clinical or pathological abnormality observed at a dose ≤100 mg/kg. Hence, the maximal tolerated single dose is >100 mg/kg.

The development of BCMA-directed CAR-Ts, antibody–drug conjugates, and bispecific antibodies have enjoyed considerable clinical success with a subset of patients reporting complete remission after treatment. Unfortunately, dose-limiting normal tissue toxicity—including cytokine storm syndrome, peripheral neuropathy, and ocular toxicity—remains a major clinical challenge in this group of treated patients. Additionally, elderly patients are often excluded from both CAR-T and antibody–drug conjugate treatments owing to their poor overall health, representing a critically unmet clinical need within this population (Caimi et al., 2021; Lonial et al., 2020; Raje et al., 2019). Therefore, new efficacious therapeutics with favorable safety and tolerability profiles are needed for treating patients ineligible for both frontline and new investigational MM and DLBCL therapies associated with high drug-related toxicity. The activation of APRIL- and BAFF-mediated signaling through BCMA, TACI, and BAFF-R provides prosurvival signals to sustain MM and DLBCL growth.

In this study, we developed a high-affinity sBCMA-Fc V3 trap for both APRIL and BAFF exhibiting superior antitumor activities in models of MM and DLBCL. More importantly, sBCMA-Fc V3 has a favorable safety profile and on-target mechanism of action in both murine and nonhuman primate models. Collectively, these data support sBCMA-Fc V3 as a clinically viable candidate for the treatment of APRIL- and BAFF-driven B cell malignancies and autoimmune disease.

## Materials and methods

### Study approval

This study was designed to characterize the therapeutic efficacy and biological functionality of engineered soluble BCMA decoy receptor as a treatment for APRIL- and BAFF-driven MM and DLBCL. MM and DLBCL patient specimens were collected from patients treated at Stanford Cancer Center under the approval of Stanford institutional review board (protocol no. 13535). Healthy blood specimens were obtained from Stanford Blood Center under the same institutional review board protocol. In vivo animal studies were conducted under the approval of AAAPLAC at Stanford University. Sample sizes for animals were determined based on previously conducted in vivo studies for power calculations. All animals were randomly assigned to treatment groups. Samples were not excluded from studies except for animals that required early termination due to unforeseeable illness unrelated to the study. Endpoints of experiments were defined in advance for each experiment. Tumor growth curves were presented for studies where tumor growth was measurable, serum levels of myeloma protein levels were used as a marker of tumor progression in MM orthotopic PDX model, and Kaplan–Meier analysis was used to define survival advantages in the PDX study; all other studies with measurable subcutaneous tumor used final tumor growth as study endpoint. Appropriate statistical analysis was used for each study.

### Primate toxicology studies

The purposes of this study were to evaluate acute toxicity after single administration of sBCMA-V3 via i.v. infusion in cynomolgus monkeys, to provide the maximum tolerated dose as reference for the design of subsequent toxicity studies and clinical trials, and to characterize the toxicokinetics and immunogenicity. This study was contracted to the Center for Drug Safety Evaluation and Research, Shanghai Institute of Materia Medica, Chinese Academy of Sciences, Shanghai, PR China. This study was approved by the Center for Drug Safety Evaluation and Research–Shanghai Institute of Materia Medica ethics committee for experimental usage and performed under the guidelines of the National Medical Products Administration: Guideline on Single Dose Toxicity Studies for Pharmaceuticals, May 2014; National Medical Products Administration: General Guideline on Non-clinical Safety Evaluation for Therapeutic Biological Products, January 2007; International Council for Harmonisation (ICH) Guideline M3 (R2): Guideline on Nonclinical Safety Studies for the Conduct of Human Clinical Trials and Marketing Authorization for Pharmaceuticals; CPMP/ICH/286/95, June 2009; and ICH S6 (R1): Guideline on Preclinical Safety Evaluation of Biotechnology-Derived Pharmaceuticals, June 2011. Justification for selection of animal species, number of animals and route of administration is as follows: (1) the cynomolgus monkey is considered an appropriate nonrodent species for safety evaluation of sBCMA-Fc V3; (2) the minimum number of animals used in this study meets the requirements of scientific evaluation of the toxicity of the test article; and (3) sBCMA-Fc V3 was administered via i.v. infusion in this study because i.v. administration is the intended administration route in humans.

### Cell lines

Human MM cell lines U266, MM1.R, and INA6 were maintained in RPMI-1640 supplemented with 10% FBS and 1% penicillin/streptomycin in standing flasks and a humidified 37°C, 5% CO_2_ incubator. INA6 cells were supplemented with 2 ng/ml human IL-6 to maintain growth. All cell lines were generously given by A.C. Koong and D. Jiang at the Department of Radiation Oncology, MD Anderson Cancer Center (Houston, TX). Isolation of B cells from healthy donors and MM patients was performed using EasySep Human B-Cell Isolation Kit according to the manufacturer’s protocol (cat. no. 17954; Stemcell Technologies). DLBCL cells SU-DHL-6 and Daudi were purchased from ATCC (cat. no. CRL-2959 and CCL-213; ATCC).

### Establishing MM PDX models using MM patient specimens

Mononuclear cells were isolated from bone marrow aspirate of MM patients and inoculated into the left tibias of 5–6-wk-old NSG mice. The injection path into the tibia was established using an empty needle penetrating through the tibia bone, guided by x ray. The empty needle was removed, and the x ray was turned off to avoid MM cell exposure to radiation. Patient cells were inoculated using a fresh needle and syringe. MM tumor growth was monitored by serum level of human IgG (M) protein. Once the host mice showed successful engraftment, marked by an increase of serum human IgG levels, the animal was sacrificed, bone marrow was flushed, and mononuclear cells were collected for intratibial injection into two host mice. This process was repeated until sufficient numbers were reached for each study. 11 patient samples were inoculated, and 2 patient samples were successfully propagated for in vivo studies.

### In vivo studies

All animal experiments were reviewed and approved by the Institutional Animal Care and Use Committee at Stanford University. Female NOD-*scid* γ mice aged 6–8 wk were purchased from the Jackson Laboratory (stock no. 005557) and used for all in vivo analysis throughout the study. Mice were housed in a pathogen-free animal facility, kept under a controlled environment with 12-h light–dark cycles. For INA6 and MM1.R tumor studies (dox-inducible BCMA KO, WT sBCMA-Fc, and sBCMA-Fc V3), 1 × 10^7^ cells were injected s.c. with 50% growth factor–reduced Matrigel (cat. no. 356230; Corning). Body weight and tumor growth were measured three times a week until study termination. Animals were terminated when the subcutaneous tumor reached the ethical termination point. For PDX studies, nonterminal bleeding was performed on animals every 14 d for evaluating serum M protein as a marker of tumor progression. Animals were terminated at signs of physical distress. For DLBCL tumor studies, female NOD-*scid* γ mice aged 6–8 wk were purchased from the Jackson Laboratory (stock no. 001303). Mice were housed in the same pathogen-free animal facility as mice for MM studies. For SU-DHL-6 and Daudi tumor studies, 5 × 10^6^ cells were injected subcutaneously with 50% growth factor–reduced Matrigel. Body weight and tumor growth were measured once a week until study termination. Wild-type sBCMA-Fc and sBCMA-Fc V3 were manufactured by ChemPartner Shanghai using HEK293 transient expression and a protein G purification system. Purified material was assessed by size exclusion chromatography HPLC and SDS-PAGE for quality control. αCD38 antibody (cat. no. A2027) was purchased from SelleckChem, recombinant mouse soluble TACI-Fc (cat. no. 577708) from BioLegend, and αBAFF antibody from Invitrogen (cat. no. MA1-822774; Thermo Fisher Scientific).

### Ribo-seq RNA library preparation

Briefly, snap-frozen cell pellets (100 million cells per sample) were lysed in polysome lysis buffer (20 mM Tris-HCl, pH 7.5, 250 mM NaCl, 15 mM MgCl_2_, 1 mM dithiothreitol, 0.5% Triton X-100, 0.024 U/ml TurboDNase, 0.48 U/ml RNasin, and 0.1 mg/ml cycloheximide [CHX]). Lysates were centrifuged for 10 min at 4°C, 14,000 *g*. The supernatant was used for isolation of ribosome-bound mRNA and total mRNA-seq. SUPERase-In (0.24 U/ml) was added to the lysate used for polysome fractionation to prevent RNA degradation. Library preparation was performed using SMARTer smRNA-Seq Kit for Illumina (cat. no. 635029; Takara Bio).

### Ribo-seq analysis

The sequencing files for ribosome profiling and RNA-seq data were processed using RiboFlow (Ozadam et al., 2020). All source code is freely available at https://github.com/ribosomeprofiling. Briefly, the 3′ adapter sequence (AAAAAAAAAA) was removed from all reads using cutadapt. The 5′ end of each read includes three bases from the template switching reaction and was also removed before alignment. We used a sequential alignment strategy to first filter out rRNA and tRNA mapping reads, followed by mapping to representative isoforms for each as defined in the APPRIS database (Rodriguez et al., 2018). Next, PCR duplicates were removed from the ribosome profiling data using the 5′ end of the sequence alignment coordinates. Finally, the resulting information was compiled into a ribo file (Ozadam et al., 2020) for downstream analyses. Data obtained from Ribo-seq analysis can be accessed through the GEO repository with accession number GSE206045.

### Bioinformatic analysis

All statistical analyses were carried out using RiboR (Ozadam et al., 2020). For quantification of ribosome occupancy, footprints of length 26 to 30 nucleotides inclusive were used. Metagene plots were generated using the 5′ end of each ribosome footprint. Ribosome occupancy and RNA-seq data were jointly analyzed, and transcript specific dispersion estimates were calculated after trimmed mean of the M-value (TMM) normalization (Robinson and Oshlack, 2010). To identify genes with differential translation efficiency, we used a generalized linear model that treats RNA expression and ribosome occupancy as two experimental manipulations of the RNA pool of the cells as previously described (Cenik et al., 2015). The model was fitted using edgeR (Robinson and Oshlack, 2010), and P values were adjusted for multiple hypothesis testing using Benjamini–Hochberg correction.

We used an adjusted P value threshold of 0.05 to define significant differences. R packages cowplot, pheatmap, EnhancedVolcano, ggpubr, ggplot2, and reshape2 were used for analyses and plotting (Kassambara and Moreaux, 2018).

### Polysome analysis

U266 cells were transfected with siScramble and siBCMA as described. Cells were pelleted and lysed in buffer (20 mM Tris-HCl, pH 7.4, 100 mM NaCl, 5 mM MgCl_2_, 1 mM dithiothreitol, 1% Triton X-100, 0.1% NP-40, 100 μg/ml CHX, 20 U/ml TurboDNase I, and complete protease inhibitor EDTA-free) in nuclease-free water. After lysis, RNA concentrations were measured using a Nanodorop UV spectrophotometer, and normalized amounts of RNA were layered onto a sucrose gradient (25–50% sucrose [wt/vol], 20 mM Tris-HCl, pH 7.4, 100 mM NaCl, 15 mM MgCl_2_, and 100 μg/ml CHX) in nuclease-free water and centrifuged in a SW41Ti rotor (Beckman) for 2.5 h at 40,000 rpm at 4°C. 16 fractions were collected by the Density Gradient Fraction System (Brandel). To each fraction, 0.1 ml of 10% SDS was added and mixed. 0.1 ml of 3 M NaOAc, pH 5.5, and 0.1 ml of water were added to each SDS-containing fraction. For normalization, 500 pg of bicistronic firefly luciferase mRNA was added to each fraction.

Total RNA from each fraction was extracted using acid-phenol:chloroform. Briefly, to each fraction, 900 μl of acid-phenol:chloroform was added and mixed thoroughly. The mixture was heated at 65°C for 5 min and centrifuged at 21,000 *g* for 10 min at room temperature. The aqueous phase (700 μl) was removed and precipitated overnight at −80°C with 700 μl isopropanol and 1.5 μl GlycoBlue Coprecipitate (Invitrogen). The samples were centrifuged at 21,000 *g*, 30 min, 4°C; the supernatant was discarded; and the RNA pellet was washed twice with 500 μl of cold 75% ethanol. Pellets were dried for 15 min at room temperature and resuspended in nuclease-free water. Total RNA from each fraction was treated with 2 U/μl TurboDNase I and incubated at 37°C for 30 min, and the column was purified using RNA Clean and Concentrator-5 (Zymo) according to the manufacturer’s instructions. RNA was eluted twice in 6 μl. Except for the first fraction (F1), RNA was pooled from every three subsequent fractions (F2–4, F5–7, F8–10, F11–13, and F14–16) and measured using Nanodorop UV spectrophotometer.

For quantitative RT-PCR, 600 ng of purified RNA was used for reverse transcription with the iScript supermix (Bio-Rad) according to the manufacturer’s instructions. See RT-PCR protocol for primer sequences.

### Synthesis of yeast-displayed sBCMA library

DNA encoding human BCMA extracellular domain, amino acids Met1–Ala54, was cloned into the pCT yeast display plasmid using NheI and BamHI restriction sites. An error-prone library was created using the BCMA extracellular domain DNA as a template, and mutations were introduced by using low-fidelity Taq polymerase (Invitrogen, Thermo Fisher Scientific) and the nucleotide analogs 8-oxo-dGTP and dPTP (TriLink Biotech). Six separate PCR reactions were performed in which the concentrations of analogs and the numbers of cycles were varied to obtain a range of mutation frequencies: five cycles (200 μM), 10 cycles (2, 20, or 200 μM), and 20 cycles (2 or 20 μM). Products from these reactions were amplified using forward and reverse primers, each with 50-bp homology to the pCT plasmid in the absence of nucleotide analogs. Amplified DNA was purified using gel electrophoresis, and the pCT plasmid was digested with NheI and BamHI. Purified mutant cDNA and linearized plasmids were electroporated in a 5:1 ratio by weight into EBY100 yeast, where they were assembled in vivo through homologous recombination. Library size was estimated to be 2 × 10^8^ by dilution plating.

### Library screening

Yeast displaying high-affinity BCMA mutants were isolated from the library using FACS. For FACS round 1, equilibrium binding sorts were performed in which yeast were incubated at room temperature in PBS with 0.1% BSA (PBSA) with 2 nM APRIL (PeproTech) for 24 h. After incubation with APRIL, yeast were pelleted, washed, and resuspended in PBSA with a 1:100 mixture of anti-c-Myc FITC antibody (Abcam) and anti-HA AF647 (Invitrogen) for 1 h at 4°C. Yeast were then washed, pelleted, and resuspended using PBSA followed by FACS analysis. For FACS rounds 2–6, kinetic off-rate sorts were conducted in which yeast were incubated with 2 nM APRIL for 3 h at room temperature, washed twice to remove excess unbound APRIL, and resuspended in PBSA containing an ∼50-fold molar excess of BCMA to render unbinding events irreversible. The length of the unbinding step was as follows: sort 2, 48 h; sorts 3, 4, and 5, 72 h; and sort 6, 84 h, with all unbinding reactions performed at room temperature. During the last hour of the dissociation reaction, cells were mixed with a 1:100 mixture of anti-c-Myc FITC antibody (Abcam) and anti-HA AF647 (Invitrogen) for 1 h at 4°C. Yeast were pelleted, washed, and resuspended in 0.1% BSA. Labeled yeast were sorted by FACS using a Vantage SE flow cytometer (Stanford FACS Core Facility) and CellQuest software (Becton Dickinson). Sorts were conducted such that the 1–3% of clones with the highest APRIL binding/c-Myc expression ratio were selected, enriching the library for clones with the highest binding affinity to APRIL. In sort 1, 10^8^ cells were screened, and subsequent rounds analyzed a minimum of 10-fold the number of clones collected in the prior sort round to ensure adequate sampling of the library diversity. Selected clones were propagated and subjected to further rounds of FACS. After sorts 3, 4, 5, and 6, plasmid DNA was recovered using a Zymoprep kit (Zymo Research Corp.), transformed into DH5a supercompetent cells, and isolated using plasmid miniprep kit (Qiagen). Sequencing was performed by MCLAB.

Analysis of yeast-displayed sort products was performed using the same reagents and protocols and described for the library sorts. Samples were analyzed on a FACS Calibur (BD Biosciences), and data were analyzed using FlowJo software (TreeStar).

### Binding affinity assay

Cells were cultured in standard tissue culture conditions. Cells were harvested, and the supernatant was discarded then dispensed onto a staining plate at 3 × 10^5^ cells per well. The plate was centrifuged at 300 *g* at 4°C for 5 min. Various concentrations of sBCMA mutants and negative control were diluted in FACS buffer containing 2% FBS, 100 μl/well. Cells were incubated for 1 h at 4°C and washed twice with 200 μl FACS buffer and centrifuged at 300 *g* for 5 min. The supernatant was discarded before and after each wash. Cells were resuspended at 100 μl/well with 1:1,000 diluent with anti-human IgG-Alexa Fluor 488 (cat. no. A28175; Thermo Fisher Scientific). Plates were incubated for 1 h at 4°C. Cells were washed twice with FACS buffer and centrifuged at 300 *g* for 5 min. Supernatant was discarded, and cells were resuspended in 100 μl cold PBS. The cells were kept in the dark, and FACS analysis was carried out on a FACS CantoII (BD Biosciences). The geometric mean (measure of binding affinity) of the double-positive population was determined by using FlowJo software. To determine the *K*_*D*_ (ligand concentration that binds to half the receptor sites at equilibrium) of the binding reaction, binding affinity was plotted against ligand concentration, and the graph was analyzed using one site–specific binding in GraphPad Prism to get the *K*_*D*_ value.

### Computational structural simulation

Computation-based structural simulation was carried out using a number of structural prediction software programs. sBCMA V3 in cocomplex with hAPRIL and hBAFF was modeled using a combination of Prime from Schrödinger Suites 2021–2, Alphafold2, RoseTTAFold, trRosetta, and RosettaRemodel based on sequences and structural alignment mapped to PDB 1XU2. Mutation-mediated changes within sBCMA V3 binding to hAPRIL and BAFF was calculated by Residue Scanning Calculation Module from Bioluminate (Schrödinger). Surface complementarity and protein–protein interaction between sBCMA V3 and hAPRIL/hBAFF was calculated by Protein Interaction Analysis module from Bioluminate.

### In vitro cell-based viability assays

Cell viability was determined with a cell counting hemocytometer or Beckman Coulter counter, depending on the study. Cells were plated in 96-well plates at a density of 2,500 cells (U266, SU-DHL-6, and Daudi) or 3,000 cells (MM1.R and INA-6). For wild-type sBCMA-Fc and sBCMA-Fc V3 treatment, cells were cultured in 1% FCS RPMI-1640 overnight followed by 1 h of 100-ng recombinant APRIL or BAFF stimulation, and increasing doses of sBCMA were added to designated wells. Both APRIL and sBCMA-Fc were replenished every 48 h until experiment end on day 7.

### Mouse CT scan to confirm MM-induced bone degradation

High-resolution micro-CT images were acquired using an in vivo micro-CT scanner, SkyScan 1276 (Bruker) under isoflurane anesthesia. The scanning mode was set as 360° step-and-shoot scanning without average framing. After each scan, the projection images were reconstructed using software (NRecon with GPU acceleration; Bruker), followed by converting the set of reconstructed slices to DICOM files (DICOM converter; Bruker).

### ELISA

Serum and cell lysate expression of human APRIL (cat. no. DY884B; R&D Systems), human BAFF (cat. no. DBLYS0B; R&D Systems), mouse APRIL (cat. no. MBS738004; My BioSource), mouse BAFF (cat. no. MBLYS0; R&D Systems), human total IgG (M) protein (cat. no. BMS2091; Thermo Fisher Scientific), mouse total IgG (M) protein (cat. no. 88-50400-88; Thermo Fisher Scientific), and mouse IgM and IgA (cat. no. MBS564075 and MBS564073; My Biosources) was detected using commercial ELISA kits according to the manufacturers’ protocol.

### Immunohistochemistry

Tissues were fixed in 10% neutral buffered formalin and embedded in paraffin blocks for cutting and mounting on glass slides. For Ki67 staining, slides were deparaffined, and antigen retrieval was carried out using 10 mM citric acid buffer and 0.05% Tween 20, pH 6 Slides were removed from buffer, cooled at room temperature for 15 min, and quenched in endogenous peroxidase with 1:10 dilution of 34% hydrogen peroxide and water for 15 min. Avidin and biotin blockers were added for 15 min each. Protein block using 2% FCS was added for 20 min. The serum and antibody were diluted in PBT (1× PBS, 0.1% BSA, 0.2%, and 0.01% Tween 20). Anti-human Ki67 antibody (cat. no. sc-23900; Santa Cruz Biotechnologies) incubated overnight at 4°C. Biotinylated anti-mouse secondary antibody 1:2,500 (cat. no. BA92001; Vector Laboratories) was added on each slide and incubated at 37°C for 30 min and then incubated with STREP-HRP for 30 min at 37°C. Signals were developed using DAB substrate kit (#34002; Thermo Fisher Scientific). TUNEL apoptosis assay was carried out using ApopTag Peroxidase In Situ Apoptosis Detection Kit (cat. no. S7100; Millipore Sigma) and performed according to the manufacturer’s instructions. All cases were scanned at 40× magnification using the Leica Aperio AT2 Digital Pathology Scanner (Leica Biosystem). Images were analyzed using NDP.view2 image analysis software developed by Hamamatsu Japan.

### Immunoblotting

Cell lysates were subjected to SDS-PAGE, followed by transfer to nitrocellulose membrane. The membranes were then probed with primary Abs against total BCMA (cat. no. 27724-1-AP; Proteintech), Pan-Akt (cat. no. 4691; Cell Signaling Technology), pAkt (cat. no. 4060; Cell Signaling Technology), phospho-p38 MAPK (cat. no 4511; Cell Signaling Technology), p38 MAPK (cat. no. 8690; Cell Signaling Technology), phospho-mTOR ser2448 (cat. no. 5536; Cell Signaling Technology), total mTOR (cat. no. 2983; Cell Signaling Technology), Raptor (cat. no. 2280; Cell Signaling Technology), Phospho-Raptor Ser792 (cat. no. 89146; Cell Signaling Technology), phospho-p70 S6K (cat. no. 9204; Cell Signaling Technology), phospho-S6 (cat. no. 9204; Cell Signaling Technology), ATMINASCIZ (cat. no. AB3271-I; Millipore Sigma), and β-actin (cat. no. sc-47778 HRP; Santa Cruz Biotechnology) at 4°C overnight. The blots were then washed and probed with HRP-conjugated anti-goat (cat. no. sc-2020; Santa Cruz Biotechnology) or HRP-conjugated anti-rabbit (cat. no. A16110; Thermo Fisher Scientific) as appropriate. The blots were developed with Bio-Rad Western C Developing Reagent (cat. no. 170-5060; Bio-Rad) and visualized with a Chemidoc digital imager (cat. no. 1708280; Bio-Rad).

### In vitro genetic knockdown studies

BCMA siRNA (cat. no. L-011217-00-0005; SMARTPool) and dox-inducible shRNA (cat. no. V3SH7669-230564302; SAMRTvector) constructs were purchased through GE Dharmacon Horizon. ATMIN CRISPR KO (sc-411076) constructs were purchased from Santa Cruz Biotechnology. For siRNA, transfection procedures were carried out using a Lonza 4D-Nucleofactor device and kits in accordance with manufacturer’s protocol. For dox-inducible BCMA knockdown cells, three shRNA sequences were tested according to the transfection protocols provided by the manufacturer. Sequences 1 and 2 showed successful knockdown of BCMA and were used for subsequent in vivo testing.

### shRNA sequences

shRNA sequences were as follows: Sh1, 5′-CAG​TCC​TGC​TCT​TTT​CCA​G-3′; Sh2, 5′-CTT​GAT​GCA​GTC​TTC​ACA​G-3′; and Sh3, 5′-AGC​CAT​GCC​CAG​GAG​ACC​T-3′.

### Real-time PCR analysis

RNA was isolated using TRIzol reagent according to the manufacturer’s instructions (Invitrogen). RNA was reverse transcribed using cDNA synthesis kit (Bio-Rad). Real-time PCR was performed as previously described. Relative expression levels of target genes were normalized against the level of GAPDH expression. Fold difference (as relative mRNA expression) was calculated by the comparative threshold count (Ct) method (2^Ct(GAPDH RNA–gene of interest)^).

### Primer sequences

Primer sequences were as follows: BCMA forward, 5′-TGT​TCT​TCT​AAT​ACT​CCT​CCT​CT-3′, and reverse, 3′-AAC​TCG​TCC​TTT​AAT​GGT​TC-5′; ATMIN forward, 5′-AAC​AGC​ACT​GCA​GTC​TCA​CA-3′, and reverse, 3′-CTG​GTC​TAG​GGA​TTG​GTT​GGT-5′; and GAPDH forward, 5′-TGC​ACC​ACC​AAC​TGC​TTA​GC-3′, and reverse, 3′-GGC​ATG​GAC​TGT​GGT​CAT​GAG-5′.

### RPPA

RPPA was performed by the MD Anderson RPPA core as described in the published protocol.

### Statistical analysis

All cell number, tumor volume, survival, and quantification of in vivo and in vitro studies were conducted using Prism software (GraphPad). ANOVA with Tukey–Kramer test was used for comparing multiple treatment groups with each other. P < 0.05 was considered significant. Repeated-measures ANOVA was used for comparing multiple treatment groups measured over time. Statistical analysis of survival curves was conducted for the survival studies. A log-rank (Mantel–Cox) test was performed to compare mean survival among groups; P ≤ 0.05 was considered statistically significant.

### Online supplemental material

Fig. S1 shows the absolute requirement of BCMA signaling activation during MM progression. Fig. S2 shows both global and specific molecular changes associated with protein translation machinery. Fig. S3 shows the in vivo efficacy of MM models treated with wild-type sBCMA-Fc and appropriate therapeutic controls. Fig. S4 shows the establishment of MM PDX models and human myeloma protein levels in patient 3 and 5 PDX MM models treated with sBCMA-Fc; also, the therapeutic efficacy of sBCMA-Fc in MM was further investigated in combination with current standard-of-care and its therapeutic potential in DLBCL. Fig. S5 shows the computational structural alignment of sBCMA V3/APRIL and sBCMA V3/BAFF cocomplex and the therapeutic efficacy of sBCMA-Fc V3 in MM and DLBCL models. Table S1 shows the demographic information and treatment status of patient samples collected for establishing the MM PDX model. Tables S2, S3, S4, and S5 list the top clones selected from rounds 3–6 of affinity-based flow cytometry sorting. Table S6, S7, S8, and S9 present the hematology analysis of male and female cynomolgus monkeys dosed with sBCMA-Fc V3.

## Supplementary Material

Table S1 shows demographic information and treatment status of patient samples collected for establishing the MM PDX model.

Table S2 shows clones selected from round 3.

Table S3 shows clones selected from round 4.

Table S4 shows clones selected from round 5.

Table S5 shows clones selected from round 6.

Table S6 shows male hematology results, part I.

Table S7 shows male hematology results, part II.

Table S8 shows female hematology results, part I.

Table S9 shows female hematology results, part II.

SourceData F2 contains original blots for Fig. 2.

SourceData F3 contains original blots for Fig. 3.

SourceData FS1 contains original blots for Fig. S1.

SourceData FS5 contains original blots for Fig. S5.

## References

[bib1] Bag-Ozbek, A., and J.S. Hui-Yuen. 2021. Emerging B-cell therapies in systemic lupus erythematosus. Ther. Clin. Risk Manag. 17:39–54. 10.2147/TCRM.S252592 33488082 PMC7814238

[bib2] Barratt, J., J.A. Tumlin, Y. Suzuki, A. Kao, A. Aydemir, Y. Zima, and G. Appel. 2020. MO039 the 24-week interim analysis results of a randomized, double-blind, placebo-controlled phase II study of atacicept in patients with IGA nephropathy and persistent proteinuria. Nephrol. Dial. Transplant. 35:gfaa140. 10.1093/ndt/gfaa140.mo039

[bib3] Becker, J.R., R. Cuella-Martin, M. Barazas, R. Liu, C. Oliveira, A.W. Oliver, K. Bilham, A.B. Holt, A.N. Blackford, J. Heierhorst, . 2018. The ASCIZ-DYNLL1 axis promotes 53BP1-dependent non-homologous end joining and PARP inhibitor sensitivity. Nat. Commun. 9:5406. 10.1038/s41467-018-07855-x 30559443 PMC6297349

[bib4] Bolkun, L., D. Lemancewicz, E. Jablonska, A. Kulczynska, U. Bolkun-Skornicka, J. Kloczko, and J. Dzieciol. 2014. BAFF and APRIL as TNF superfamily molecules and angiogenesis parallel progression of human multiple myeloma. Ann. Hematol. 93:635–644. 10.1007/s00277-013-1924-9 24141333 PMC3945232

[bib5] Bossen, C., and P. Schneider. 2006. BAFF, APRIL and their receptors: Structure, function and signaling. Semin. Immunol. 18:263–275. 10.1016/j.smim.2006.04.006 16914324

[bib6] Briones, J., J.M. Timmerman, D.M. Hilbert, and R. Levy. 2002. BLyS and BLyS receptor expression in non-Hodgkin’s lymphoma. Exp. Hematol. 30:135–141. 10.1016/s0301-472x(01)00774-3 11823048

[bib7] Burger, R., A. Guenther, F. Bakker, M. Schmalzing, S. Bernand, W. Baum, B. Duerr, G.M. Hocke, H. Steininger, E. Gebhart, and M. Gramatzki. 2001. Gp130 and ras mediated signaling in human plasma cell line INA-6: A cytokine-regulated tumor model for plasmacytoma. Hematol. J. 2:42–53. 10.1038/sj.thj.6200075 11920233

[bib8] Caimi, P.F., W. Ai, J.P. Alderuccio, K.M. Ardeshna, M. Hamadani, B. Hess, B.S. Kahl, J. Radford, M. Solh, A. Stathis, . 2021. Loncastuximab tesirine in relapsed or refractory diffuse large B-cell lymphoma (LOTIS-2): A multicentre, open-label, single-arm, phase 2 trial. Lancet Oncol. 22:790–800. 10.1016/S1470-2045(21)00139-X 33989558

[bib9] Carpenter, R.O., M.O. Evbuomwan, S. Pittaluga, J.J. Rose, M. Raffeld, S. Yang, R.E. Gress, F.T. Hakim, and J.N. Kochenderfer. 2013. B-cell maturation antigen is a promising target for adoptive T-cell therapy of multiple myeloma. Clin. Cancer Res. 19:2048–2060. 10.1158/1078-0432.CCR-12-2422 23344265 PMC3630268

[bib10] Castigli, E., S. Scott, F. Dedeoglu, P. Bryce, H. Jabara, A.K. Bhan, E. Mizoguchi, and R.S. Geha. 2004. Impaired IgA class switching in APRIL-deficient mice. Proc. Natl. Acad. Sci. USA. 101:3903–3908. 10.1073/pnas.0307348101 14988498 PMC374342

[bib11] Castigli, E., S.A. Wilson, S. Scott, F. Dedeoglu, S. Xu, K.P. Lam, R.J. Bram, H. Jabara, and R.S. Geha. 2005. TACI and BAFF-R mediate isotype switching in B cells. J. Exp. Med. 201:35–39. 10.1084/jem.20032000 15630136 PMC2212754

[bib12] Cenik, C., E.S. Cenik, G.W. Byeon, F. Grubert, S.I. Candille, D. Spacek, B. Alsallakh, H. Tilgner, C.L. Araya, H. Tang, . 2015. Integrative analysis of RNA, translation, and protein levels reveals distinct regulatory variation across humans. Genome Res. 25:1610–1621. 10.1101/gr.193342.115 26297486 PMC4617958

[bib13] Chim, C.S., S.K. Kumar, R.Z. Orlowski, G. Cook, P.G. Richardson, M.A. Gertz, S. Giralt, M.V. Mateos, X. Leleu, and K.C. Anderson. 2018. Management of relapsed and refractory multiple myeloma: Novel agents, antibodies, immunotherapies and beyond. Leukemia. 32:252–262. 10.1038/leu.2017.329 29257139 PMC5808071

[bib14] Chiu, A., W. Xu, B. He, S.R. Dillon, J.A. Gross, E. Sievers, X. Qiao, P. Santini, E. Hyjek, J.W. Lee, . 2007. Hodgkin lymphoma cells express TACI and BCMA receptors and generate survival and proliferation signals in response to BAFF and APRIL. Blood. 109:729–739. 10.1182/blood-2006-04-015958 16960154 PMC1785096

[bib15] Collier, F.C., and P. Jackson. 1953. The precipitin test for Bence-Jones protein. N. Engl. J. Med. 248:409–414. 10.1056/NEJM195303052481003 13025707

[bib16] Durer, C., S. Durer, S. Lee, R. Chakraborty, M.N. Malik, A. Rafae, M.A. Zar, A. Kamal, N. Rosko, C. Samaras, . 2020. Treatment of relapsed multiple myeloma: Evidence-based recommendations. Blood Rev. 39:100616. 10.1016/j.blre.2019.100616 31500848

[bib17] Fragioudaki, M., G. Tsirakis, C.A. Pappa, I. Aristeidou, C. Tsioutis, A. Alegakis, D.S. Kyriakou, E.N. Stathopoulos, and M.G. Alexandrakis. 2012. Serum BAFF levels are related to angiogenesis and prognosis in patients with multiple myeloma. Leuk. Res. 36:1004–1008. 10.1016/j.leukres.2012.03.012 22498341

[bib18] Fu, J., H. Shi, T. Zhan, H. Li, L. Ye, L. Xie, Z. Wang, B. Wang, and L. Zheng. 2021. BST-2/Tetherin is involved in BAFF-enhanced proliferation and survival via canonical NF-κB signaling in neoplastic B-lymphoid cells. Exp. Cell Res. 398:112399. 10.1016/j.yexcr.2020.112399 33245890

[bib19] Gordon, N.C., S. Lien, J. Johnson, H.J.A. Wallweber, T. Tran, B. Currell, M. Mathieu, C. Quan, M.A. Starovasnik, S.G. Hymowitz, and R.F. Kelley. 2010. Multiple novel classes of APRIL-specific receptor-blocking peptides isolated by phage display. J. Mol. Biol. 396:166–177. 10.1016/j.jmb.2009.11.041 19945466

[bib20] Grasset, E.K., A. Chorny, S. Casas-Recasens, C. Gutzeit, G. Bongers, I. Thomsen, L. Chen, Z. He, D.B. Matthews, M.A. Oropallo, . 2020. Gut T cell-independent IgA responses to commensal bacteria require engagement of the TACI receptor on B cells. Sci. Immunol. 5:eaat7117. 10.1126/sciimmunol.aat7117 32737068 PMC8349226

[bib21] Greenstein, S., N.L. Krett, Y. Kurosawa, C. Ma, D. Chauhan, T. Hideshima, K.C. Anderson, and S.T. Rosen. 2003. Characterization of the MM.1 human multiple myeloma (MM) cell lines: A model system to elucidate the characteristics, behavior, and signaling of steroid-sensitive and -resistant MM cells. Exp. Hematol. 31:271–282. 10.1016/s0301-472x(03)00023-7 12691914

[bib22] Hatzoglou, A., J. Roussel, M.F. Bourgeade, E. Rogier, C. Madry, J. Inoue, O. Devergne, and A. Tsapis. 2000. TNF receptor family member BCMA (B cell maturation) associates with TNF receptor-associated factor (TRAF) 1, TRAF2, and TRAF3 and activates NF-κ B, elk-1, c-Jun N-terminal kinase, and p38 mitogen-activated protein kinase. J. Immunol. 165:1322–1330. 10.4049/jimmunol.165.3.1322 10903733

[bib23] Hymowitz, S.G., D.R. Patel, H.J.A. Wallweber, S. Runyon, M. Yan, J. Yin, S.K. Shriver, N.C. Gordon, B. Pan, N.J. Skelton, . 2005. Structures of APRIL-receptor complexes: Like BCMA, TACI employs only a single cysteine-rich domain for high affinity ligand binding. J. Biol. Chem. 280:7218–7227. 10.1074/jbc.M411714200 15542592

[bib24] Ingolia, N.T., J.A. Hussmann, and J.S. Weissman. 2019. Ribosome profiling: Global views of translation. Cold Spring Harb. Perspect. Biol. 11:a032698. 10.1101/cshperspect.a032698 30037969 PMC6496350

[bib25] Ingolia, N.T., L.F. Lareau, and J.S. Weissman. 2011. Ribosome profiling of mouse embryonic stem cells reveals the complexity and dynamics of mammalian proteomes. Cell. 147:789–802. 10.1016/j.cell.2011.10.002 22056041 PMC3225288

[bib26] Jurado, S., L.A. Conlan, E.K. Baker, J.L. Ng, N. Tenis, N.C. Hoch, K. Gleeson, M. Smeets, D. Izon, and J. Heierhorst. 2012a. ATM substrate Chk2-interacting Zn^2+^ finger (ASCIZ) Is a bi-functional transcriptional activator and feedback sensor in the regulation of dynein light chain (DYNLL1) expression. J. Biol. Chem. 287:3156–3164. 10.1074/jbc.M111.306019 22167198 PMC3270970

[bib27] Jurado, S., K. Gleeson, K. O’Donnell, D.J. Izon, C.R. Walkley, A. Strasser, D.M. Tarlinton, and J. Heierhorst. 2012b. The Zinc-finger protein ASCIZ regulates B cell development via DYNLL1 and Bim. J. Exp. Med. 209:1629–1639. 10.1084/jem.20120785 22891272 PMC3428950

[bib28] Jurado, S., I. Smyth, B. van Denderen, N. Tenis, A. Hammet, K. Hewitt, J.L. Ng, C.J. McNees, S.V. Kozlov, H. Oka, . 2010. Dual functions of ASCIZ in the DNA base damage response and pulmonary organogenesis. PLoS Genet. 6:e1001170. 10.1371/journal.pgen.1001170 20975950 PMC2958817

[bib29] Kariolis, M.S., Y.R. Miao, A. Diep, S.E. Nash, M.M. Olcina, D. Jiang, D.S. Jones, S. Kapur, I.I. Mathews, A.C. Koong, . 2017. Inhibition of the GAS6/AXL pathway augments the efficacy of chemotherapies. J. Clin. Invest. 127:183–198. 10.1172/JCI85610 27893463 PMC5199716

[bib30] Kassambara, A., and J. Moreaux. 2018. Analysis of global gene expression profiles. Methods Mol. Biol. 1792:157–166. 10.1007/978-1-4939-7865-6_11 29797258

[bib31] Keats, J.J., R. Fonseca, M. Chesi, R. Schop, A. Baker, W.J. Chng, S. Van Wier, R. Tiedemann, C.X. Shi, M. Sebag, . 2007. Promiscuous mutations activate the noncanonical NF-κB pathway in multiple myeloma. Cancer Cell. 12:131–144. 10.1016/j.ccr.2007.07.003 17692805 PMC2083698

[bib32] Kuo, S.H., P.Y. Yeh, L.T. Chen, M.S. Wu, C.W. Lin, K.H. Yeh, Y.S. Tzeng, J.Y. Chen, P.N. Hsu, J.T. Lin, and A.L. Cheng. 2008. Overexpression of B cell-activating factor of TNF family (BAFF) is associated with Helicobacter pylori-independent growth of gastric diffuse large B-cell lymphoma with histologic evidence of MALT lymphoma. Blood. 112:2927–2934. 10.1182/blood-2008-02-137513 18628489

[bib33] Lee, W.S., and O. Amengual. 2020. B cells targeting therapy in the management of systemic lupus erythematosus. Immunol. Med. 43:16–35. 10.1080/25785826.2019.1698929 32107989

[bib34] Leszczynska, K.B., E.L. Gottgens, D. Biasoli, M.M. Olcina, J. Ient, S. Anbalagan, S. Bernhardt, A.J. Giaccia, and E.M. Hammond. 2016. Mechanisms and consequences of ATMIN repression in hypoxic conditions: Roles for p53 and HIF-1. Sci. Rep. 6:21698. 10.1038/srep21698 26875667 PMC4753685

[bib35] Liu, Y., X. Hong, J. Kappler, L. Jiang, R. Zhang, L. Xu, C.H. Pan, W.E. Martin, R.C. Murphy, H.B. Shu, . 2003. Ligand-receptor binding revealed by the TNF family member TALL-1. Nature. 423:49–56. 10.1038/nature01543 12721620

[bib36] Lonial, S., H.C. Lee, A. Badros, S. Trudel, A.K. Nooka, A. Chari, A.O. Abdallah, N. Callander, N. Lendvai, D. Sborov, . 2020. Belantamab mafodotin for relapsed or refractory multiple myeloma (DREAMM-2): A two-arm, randomised, open-label, phase 2 study. Lancet Oncol. 21:207–221. 10.1016/S1470-2045(19)30788-0 31859245

[bib37] Lyu, M.A., D. Rai, K.S. Ahn, B. Sung, L.H. Cheung, J.W. Marks, B.B. Aggarwal, R.C.T. Aguiar, V. Gandhi, and M.G. Rosenblum. 2010. The rGel/BLyS fusion toxin inhibits diffuse large B-cell lymphoma growth in vitro and in vivo. Neoplasia. 12:366–375. 10.1593/neo.91960 20454508 PMC2864474

[bib38] Marsters, S.A., M. Yan, R.M. Pitti, P.E. Haas, V.M. Dixit, and A. Ashkenazi. 2000. Interaction of the TNF homologues BLyS and APRIL with the TNF receptor homologues BCMA and TACI. Curr. Biol. 10:785–788. 10.1016/s0960-9822(00)00566-2 10898980

[bib39] McNees, C.J., L.A. Conlan, N. Tenis, and J. Heierhorst. 2005. ASCIZ regulates lesion-specific Rad51 focus formation and apoptosis after methylating DNA damage. EMBO J. 24:2447–2457. 10.1038/sj.emboj.7600704 15933716 PMC1173145

[bib40] Miao, Y.R., K.N. Thakkar, J. Qian, M.S. Kariolis, W. Huang, S. Nandagopal, T.T.C. Yang, A.N. Diep, G.M. Cherf, Y. Xu, . 2021. Neutralization of PD-L2 is essential for overcoming immune checkpoint blockade resistance in ovarian cancer. Clin. Cancer Res. 27:4435–4448. 10.1158/1078-0432.CCR-20-0482 34011561 PMC8338886

[bib41] Moreaux, J., D. Hose, M. Jourdan, T. Reme, M. Hundemer, M. Moos, N. Robert, P. Moine, J. De Vos, H. Goldschmidt, and B. Klein. 2007. TACI expression is associated with a mature bone marrow plasma cell signature and C-MAF overexpression in human myeloma cell lines. Haematologica. 92:803–811. 10.3324/haematol.10574 17550853 PMC2789280

[bib42] Moreaux, J., E. Legouffe, E. Jourdan, P. Quittet, T. Reme, C. Lugagne, P. Moine, J.F. Rossi, B. Klein, and K. Tarte. 2004. BAFF and APRIL protect myeloma cells from apoptosis induced by interleukin 6 deprivation and dexamethasone. Blood. 103:3148–3157. 10.1182/blood-2003-06-1984 15070697 PMC2387243

[bib43] O’Connor, B.P., V.S. Raman, L.D. Erickson, W.J. Cook, L.K. Weaver, C. Ahonen, L.L. Lin, G.T. Mantchev, R.J. Bram, and R.J. Noelle. 2004. BCMA is essential for the survival of long-lived bone marrow plasma cells. J. Exp. Med. 199:91–98. 10.1084/jem.20031330 14707116 PMC1887725

[bib44] Ozadam, H., M. Geng, and C. Cenik. 2020. RiboFlow, RiboR and RiboPy: An ecosystem for analyzing ribosome profiling data at read length resolution. Bioinformatics. 36:2929–2931. 10.1093/bioinformatics/btaa028 31930375 PMC7203755

[bib45] Pelletier, M., J.S. Thompson, F. Qian, S.A. Bixler, D. Gong, T. Cachero, K. Gilbride, E. Day, M. Zafari, C. Benjamin, . 2003. Comparison of soluble decoy IgG fusion proteins of BAFF-R and BCMA as antagonists for BAFF. J. Biol. Chem. 278:33127–33133. 10.1074/jbc.M305754200 12796483

[bib46] Pham, L.V., L. Fu, A.T. Tamayo, C. Bueso-Ramos, E. Drakos, F. Vega, L.J. Medeiros, and R.J. Ford. 2011. Constitutive BR3 receptor signaling in diffuse, large B-cell lymphomas stabilizes nuclear factor-κB-inducing kinase while activating both canonical and alternative nuclear factor-κB pathways. Blood. 117:200–210. 10.1182/blood-2010-06-290437 20889926 PMC3037744

[bib47] Raje, N., J. Berdeja, Y. Lin, D. Siegel, S. Jagannath, D. Madduri, M. Liedtke, J. Rosenblatt, M.V. Maus, A. Turka, . 2019. Anti-BCMA CAR T-cell therapy bb2121 in relapsed or refractory multiple myeloma. N. Engl. J. Med. 380:1726–1737. 10.1056/NEJMoa1817226 31042825 PMC8202968

[bib48] Rapali, P., M.F. Garcia-Mayoral, M. Martinez-Moreno, K. Tarnok, K. Schlett, J.P. Albar, M. Bruix, L. Nyitray, and I. Rodriguez-Crespo. 2011. LC8 dynein light chain (DYNLL1) binds to the C-terminal domain of ATM-interacting protein (ATMINASCIZ) and regulates its subcellular localization. Biochem. Biophys. Res. Commun. 414:493–498. 10.1016/j.bbrc.2011.09.093 21971545

[bib49] Robinson, M.D., and A. Oshlack. 2010. A scaling normalization method for differential expression analysis of RNA-seq data. Genome Biol. 11:R25. 10.1186/gb-2010-11-3-r25 20196867 PMC2864565

[bib50] Rodriguez, J.M., J. Rodriguez-Rivas, T. Di Domenico, J. Vazquez, A. Valencia, and M.L. Tress. 2018. APPRIS 2017: Principal isoforms for multiple gene sets. Nucleic Acids Res. 46:D213–D217. 10.1093/nar/gkx997 29069475 PMC5753224

[bib51] Rossi, J.F., J. Moreaux, D. Hose, G. Requirand, M. Rose, V. Rouille, I. Nestorov, G. Mordenti, H. Goldschmidt, A. Ythier, and B. Klein. 2009. Atacicept in relapsed/refractory multiple myeloma or active Waldenstrom’s macroglobulinemia: A phase I study. Br. J. Cancer. 101:1051–1058. 10.1038/sj.bjc.6605241 19789533 PMC2768101

[bib52] Samy, E., S. Wax, B. Huard, H. Hess, and P. Schneider. 2017. Targeting BAFF and APRIL in systemic lupus erythematosus and other antibody-associated diseases. Int. Rev. Immunol. 36:3–19. 10.1080/08830185.2016.1276903 28215100

[bib53] Schuepbach-Mallepell, S., D. Das, L. Willen, M. Vigolo, A. Tardivel, L. Lebon, C. Kowalczyk-Quintas, J. Nys, C. Smulski, T.S. Zheng, . 2015. Stoichiometry of heteromeric BAFF and APRIL cytokines dictates their receptor binding and signaling properties. J. Biol. Chem. 290:16330–16342. 10.1074/jbc.M115.661405 25953898 PMC4481231

[bib54] Shin, W., H.T. Lee, H. Lim, S.H. Lee, J.Y. Son, J.U. Lee, K.Y. Yoo, S.E. Ryu, J. Rhie, J.Y. Lee, and Y.S. Heo. 2018. BAFF-neutralizing interaction of belimumab related to its therapeutic efficacy for treating systemic lupus erythematosus. Nat. Commun. 9:1200. 10.1038/s41467-018-03620-2 29572471 PMC5865148

[bib55] Shrestha, S., P. Budhathoki, Y. Adhikari, A. Marasini, S. Bhandari, W.A.Y. Mir, and D.B. Shrestha. 2021. Belimumab in lupus nephritis: A systematic review and meta-analysis. Cureus. 13:e20440. 10.7759/cureus.20440 35047277 PMC8760003

[bib56] Swerdlow, S.H., E. Campo, S.A. Pileri, N.L. Harris, H. Stein, R. Siebert, R. Advani, M. Ghielmini, G.A. Salles, A.D. Zelenetz, and E.S. Jaffe. 2016. The 2016 revision of the World Health Organization classification of lymphoid neoplasms. Blood. 127:2375–2390. 10.1182/blood-2016-01-643569 26980727 PMC4874220

[bib57] Tai, Y.T., and K.C. Anderson. 2019. B cell maturation antigen (BCMA)-based immunotherapy for multiple myeloma. Expert Opin. Biol. Ther. 19:1143–1156. 10.1080/14712598.2019.1641196 31277554 PMC6785394

[bib58] Weber, T., and R. Schmitz. 2022. Molecular subgroups of diffuse large B cell lymphoma: Biology and implications for clinical practice. Curr. Oncol. Rep. 24:13–21. 10.1007/s11912-021-01155-2 35060000 PMC8831345

[bib59] Wu, Y., D. Bressette, J.A. Carrell, T. Kaufman, P. Feng, K. Taylor, Y. Gan, Y.H. Cho, A.D. Garcia, E. Gollatz, . 2000. Tumor necrosis factor (TNF) receptor superfamily member TACI is a high affinity receptor for TNF family members APRIL and BLyS. J. Biol. Chem. 275:35478–35485. 10.1074/jbc.M005224200 10956646

[bib60] Yu, G., T. Boone, J. Delaney, N. Hawkins, M. Kelley, M. Ramakrishnan, S. McCabe, W.R. Qiu, M. Kornuc, X.Z. Xia, . 2000. APRIL and TALL-I and receptors BCMA and TACI: System for regulating humoral immunity. Nat. Immunol. 1:252–256. 10.1038/79802 10973284

