# Supplementary material for: Developing high-affinity decoy receptors to treat multiple myeloma and diffuse large B cell lymphoma
Source: J Exp Med. 2022 Jul 26;219(9):e20220214. doi: 10.1084/jem.20220214 (PMC9428257; doi:10.1084/jem.20220214)
Supplement: Table S1 — shows demographic information and treatment status of patient samples collected for establishing the MM PDX model. [file JEM_20220214_TableS1.docx]

**Table S1.** Demographic information and treatment status of patient samples collected for establishing multiple myeloma PDX model.

**
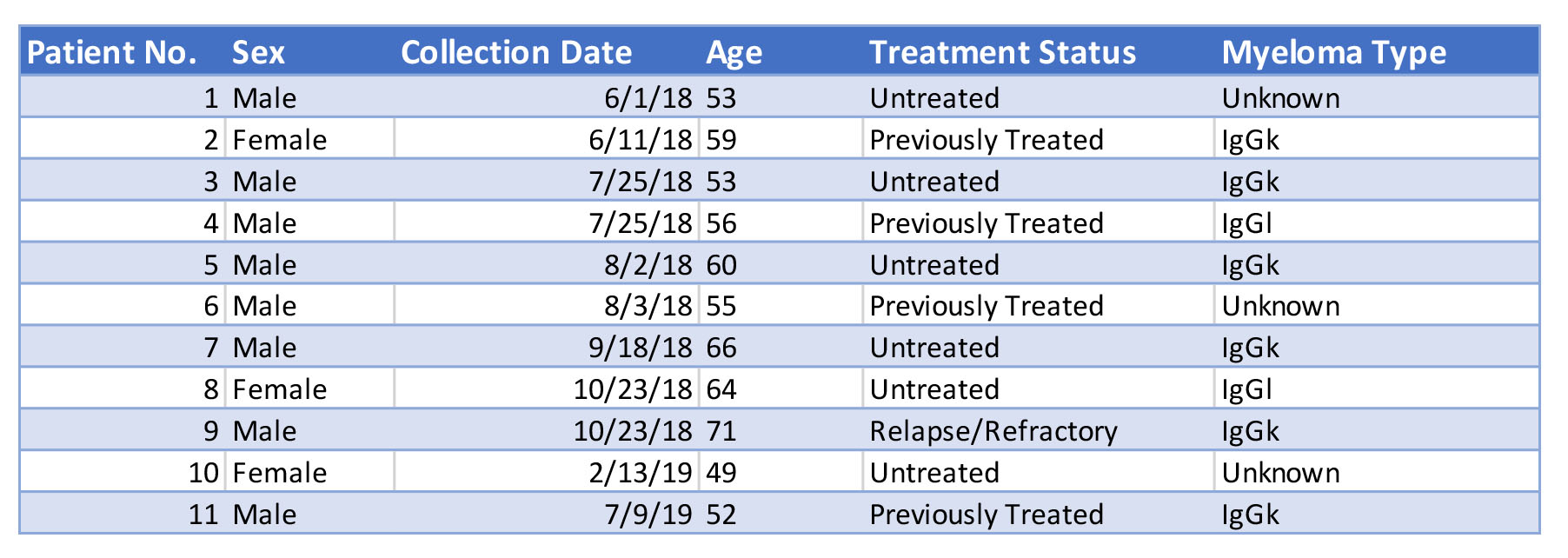
**
