# Supplementary figures and images for: Developing high-affinity decoy receptors to treat multiple myeloma and diffuse large B cell lymphoma
Source: J Exp Med. 2022 Jul 26;219(9):e20220214. doi: 10.1084/jem.20220214 (PMC9428257; doi:10.1084/jem.20220214)

**Table S2.** Clones selected from round 3

**
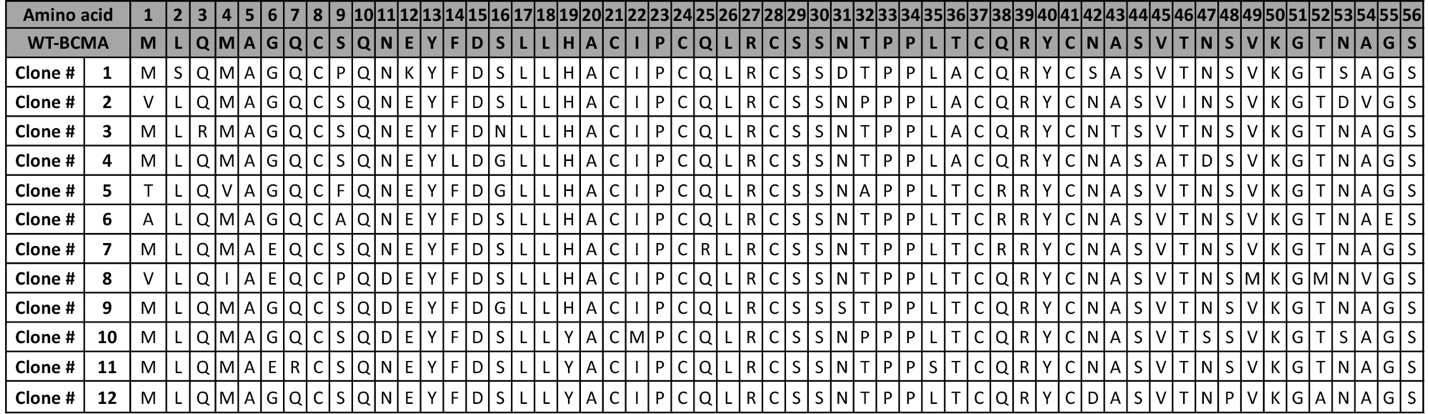
**

Supplement: Table S2 — shows clones selected from round 3. [file JEM_20220214_TableS2.docx]

**Table S3.** Clones selected from round 4


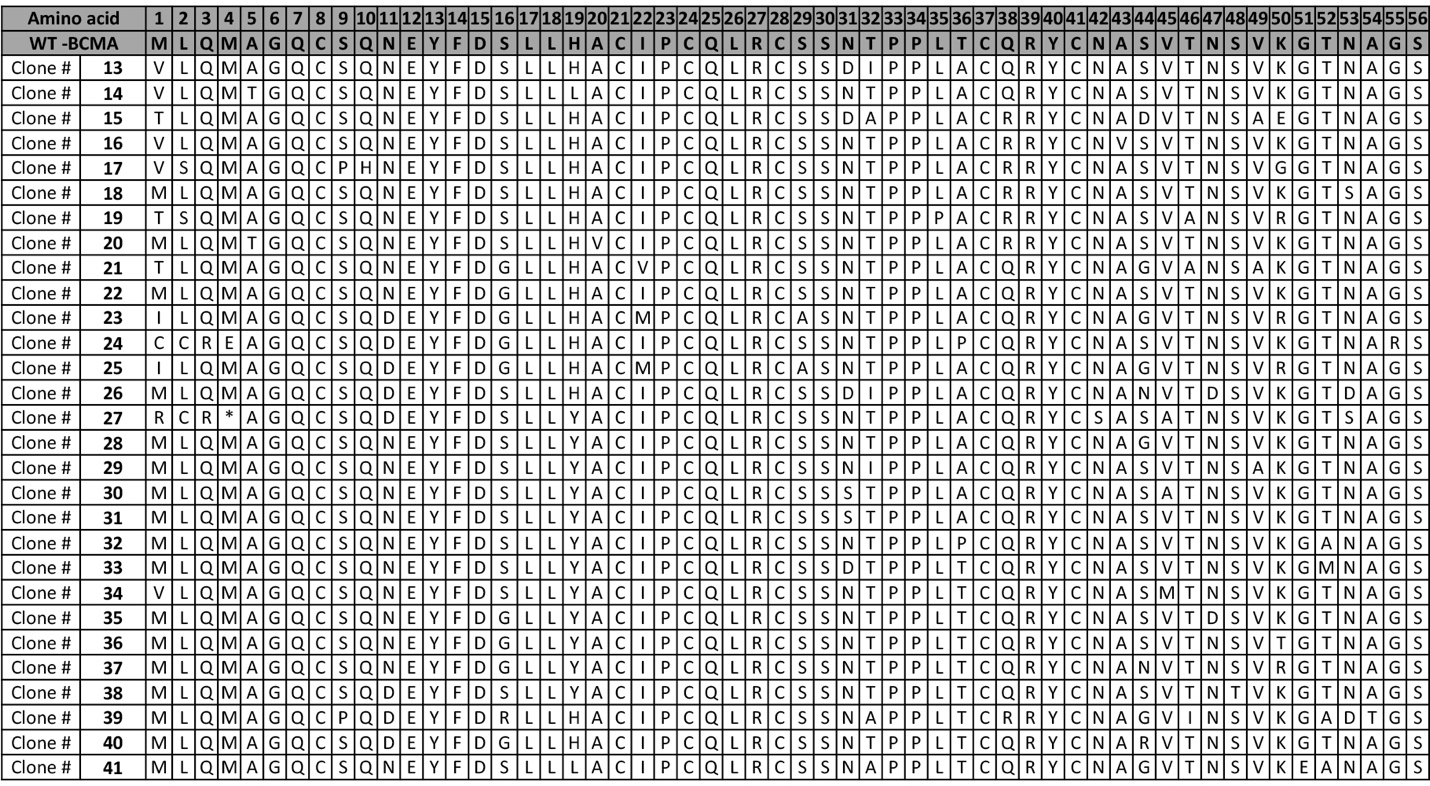

Supplement: Table S3 — shows clones selected from round 4. [file JEM_20220214_TableS3.docx]

**Table S4.** Clones selected from round 5

**
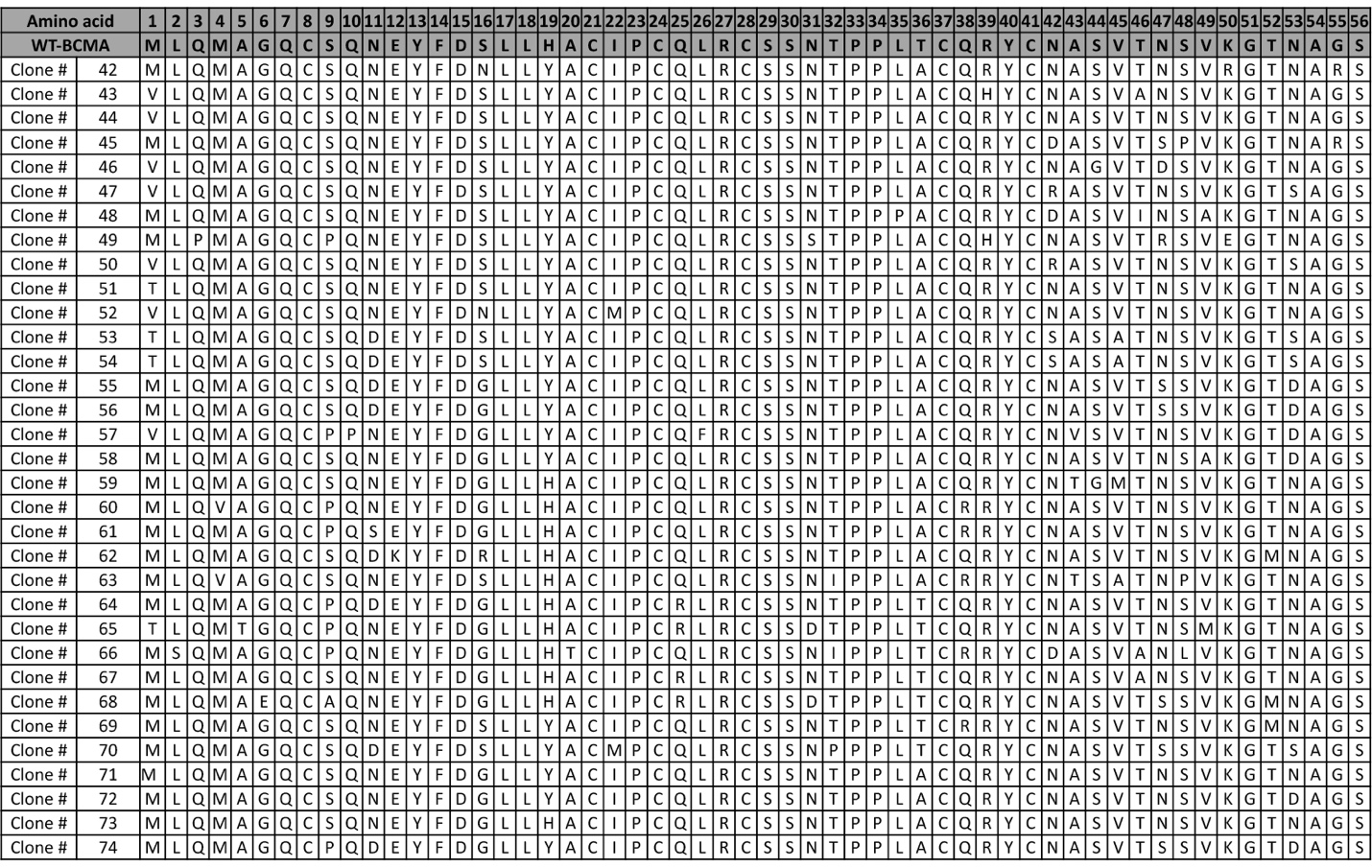
**

Supplement: Table S4 — shows clones selected from round 5. [file JEM_20220214_TableS4.docx]

**Table S5.** Clones selected from round 6


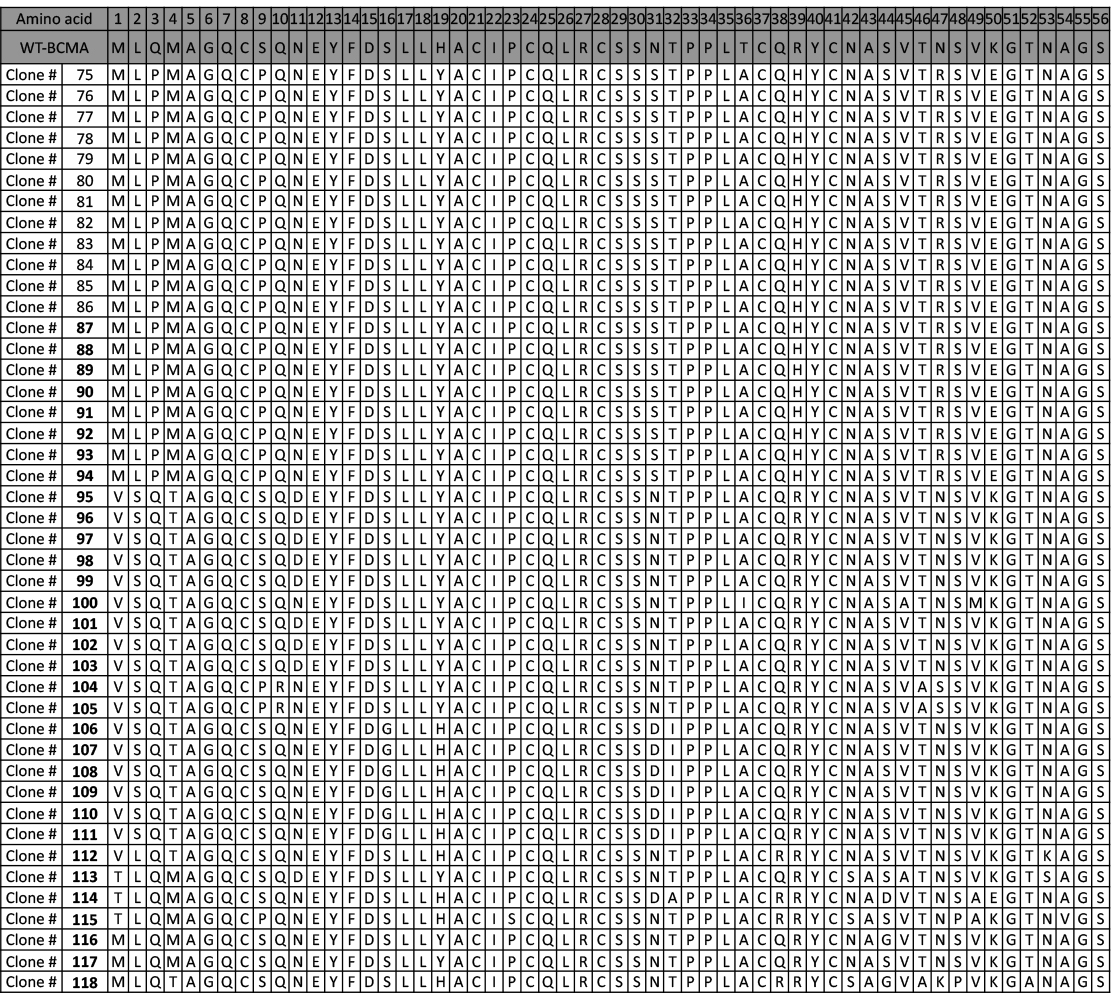

Supplement: Table S5 — shows clones selected from round 6. [file JEM_20220214_TableS5.docx]

## SourceDataF2A

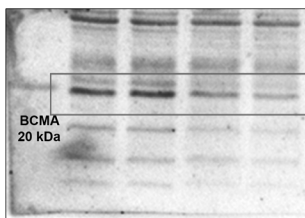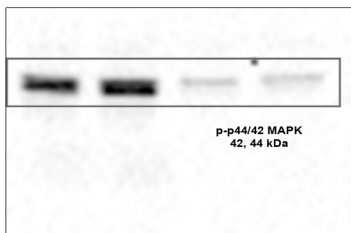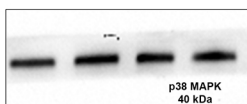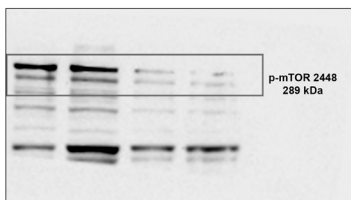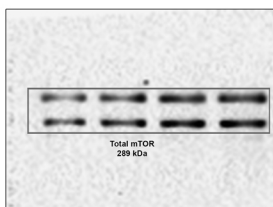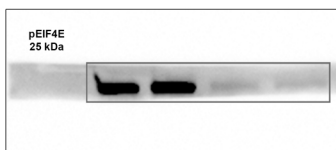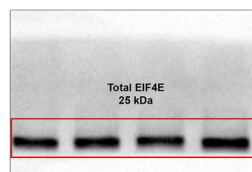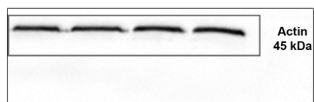

## SourceData F2I

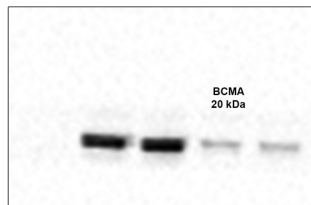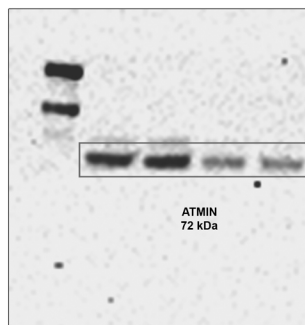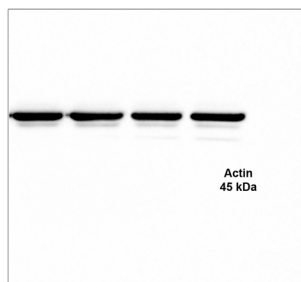

Supplement: SourceData F2 — contains original blots for Fig. 2. [file JEM_20220214_SourceDataF2.pdf]

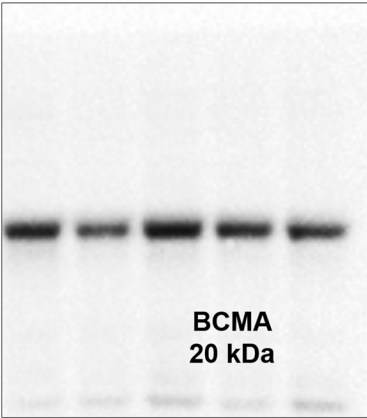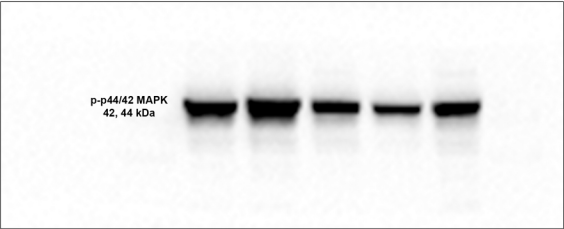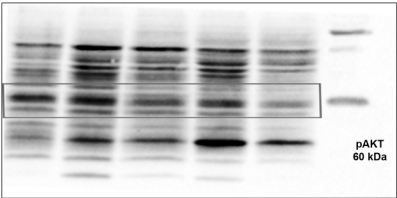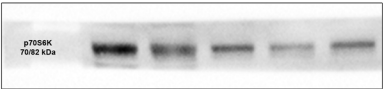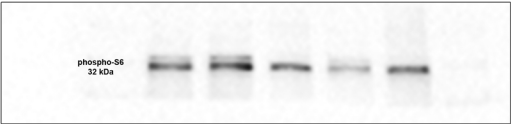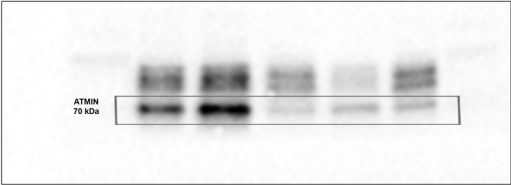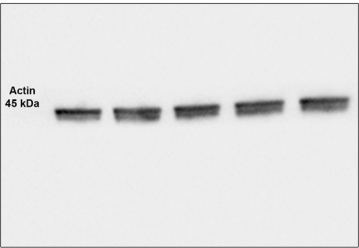

Supplement: SourceData F3 — contains original blots for Fig. 3. [file JEM_20220214_SourceDataF3.pdf]

## SourceData FS1B

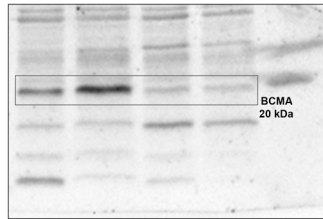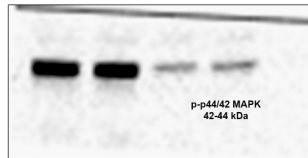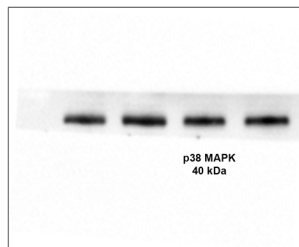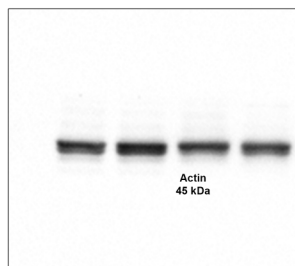

Supplement: SourceData FS1 — contains original blots for Fig. S1. [file JEM_20220214_SourceDataFS1.pdf]

## SourceData FS5G

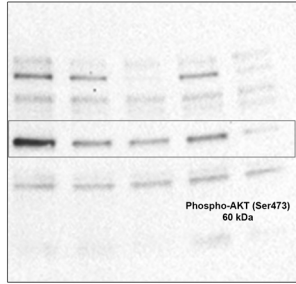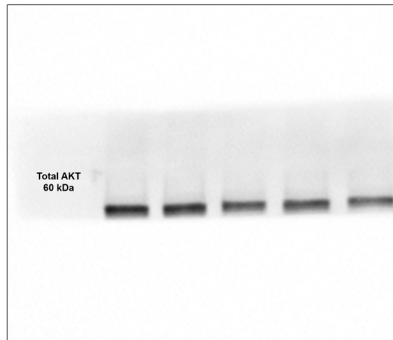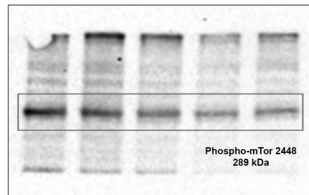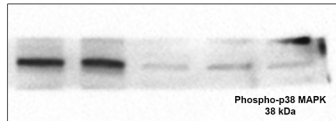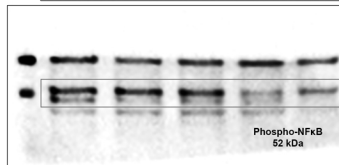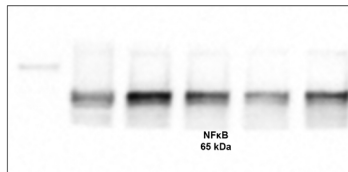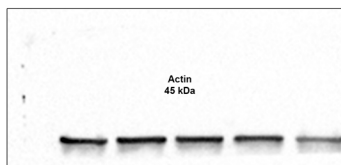

Supplement: SourceData FS5 — contains original blots for Fig. S5. [file JEM_20220214_SourceDataFS5.pdf]
