# Supplementary material for: Developing high-affinity decoy receptors to treat multiple myeloma and diffuse large B cell lymphoma
Source: J Exp Med. 2022 Jul 26;219(9):e20220214. doi: 10.1084/jem.20220214 (PMC9428257; doi:10.1084/jem.20220214)
Supplement: Table S6 — shows male hematology results, part I. [file JEM_20220214_TableS6.docx]

**Table S6.** Male hematology results, part I

| Vehiclecontrol | Day(s) relative to start date |  |  |  |  |  |  |  |  |
| --- | --- | --- | --- | --- | --- | --- | --- | --- | --- |
|  |  | RBC  (10^12^/liter) | HGB  (g/liter) | HCT  (%) | MCV  (fl) | MCH  (pg) | MCHC  (g/liter) | RDW  (%) | RET  (10^9^/liter) |
| 1101 | -13 | 6.21 | 139 | 49.4 | 79.6 | 22.4 | 281 | 13.3 | 192.0 |
|  | -6 | 5.89 | 133 | 47.4 | 80.6 | 22.6 | 280 | 13.1 | 121.0 |
|  | 1 | 5.47 | 124 | 41.7 | 76.3 | 22.7 | 298 | 13.3 | 154.9 |
|  | 2 | 5.29 | 119 | 43.7 | 82.5 | 22.5 | 273 | 13.4 | 198.3 |
|  | 7 | 4.99 | 112 | 38.9 | 77.9 | 22.4 | 287 | 13.6 | 235.8 |
|  | 14 | 5.42 | 124 | 40.5 | 74.6 | 22.8 | 305 | 13.3 | 169.3 |
|  | 42 | 5.81 | 129 | 42.6 | 73.4 | 22.3 | 303 | 13.4 | 109.6 |

| Treatment 0.1 mg/kg | Day(s) relative to start date |  |  |  |  |  |  |  |  |
| --- | --- | --- | --- | --- | --- | --- | --- | --- | --- |
|  |  | RBC  (10^12^/liter) | HGB  (g/liter) | HCT  (%) | MCV  (fl) | MCH  (pg) | MCHC  (g/liter) | RDW  (%) | RET  (10^9^/liter) |
| 1203 | -13 | 5.61 | 126 | 43.5 | 77.6 | 22.4 | 289 | 13.5 | 209.8 |
|  | -6 | 6.23 | 140 | 47.9 | 76.8 | 22.5 | 293 | 12.9 | 140.6 |
|  | 1 | 5.61 | 126 | 42.6 | 75.9 | 22.5 | 297 | 12.7 | 136.3 |
|  | 2 | 5.12 | 114 | 39.0 | 76.2 | 22.3 | 292 | 12.9 | 157.0 |
|  | 7 | 5.18 | 116 | 38.6 | 74.6 | 22.4 | 301 | 13.1 | 236.5 |
|  | 14 | 5.55 | 122 | 40.2 | 72.4 | 22.0 | 304 | 12.9 | 178.4 |
|  | 42 | 6.14 | 139 | 44.4 | 72.3 | 22.6 | 313 | 12.1 | 89.2 |

| Treatment 1 mg/kg | Day(s) relative to start date |  |  |  |  |  |  |  |  |
| --- | --- | --- | --- | --- | --- | --- | --- | --- | --- |
|  |  | RBC  (10^12^/liter) | HGB  (g/liter) | HCT  (%) | MCV  (fl) | MCH  (pg) | MCHC  (g/liter) | RDW  (%) | RET  (10^9^/liter) |
| 1305 | -13 | 6.18 | 137 | 45.6 | 73.8 | 22.1 | 300 | 12.9 | 250.0 |
|  | -6 | 6.55 | 147 | 47.7 | 72.9 | 22.5 | 309 | 12.3 | 160.5 |
|  | -3 | 6.69 | 151 | 47.9 | 71.6 | 22.5 | 315 | 12.2 | 137.3 |
|  | 1 | 6.37 | 142 | 45.0 | 70.6 | 22.3 | 316 | 12.2 | 123.4 |
|  | 2 | 5.67 | 127 | 41.3 | 72.8 | 22.4 | 307 | 12.4 | 144.8 |
|  | 7 | 5.71 | 128 | 40.2 | 70.3 | 22.3 | 317 | 12.5 | 172.0 |
|  | 14 | 6.08 | 132 | 42.7 | 70.2 | 21.8 | 310 | 12.2 | 188.3 |
|  | 42 | 6.26 | 136 | 42.4 | 67.7 | 21.7 | 320 | 11.9 | 95.1 |

| Treatment 10 mg/kg | Day(s) relative to start date |  |  |  |  |  |  |  |  |
| --- | --- | --- | --- | --- | --- | --- | --- | --- | --- |
|  |  | RBC  (10^12^/liter) | HGB  (g/liter) | HCT  (%) | MCV  (fl) | MCH  (pg) | MCHC  (g/liter) | RDW  (%) | RET  (10^9^/liter) |
| 1407 | -13 | 5.00 | 115 | 38.1 | 76.1 | 22.9 | 301 | 13.8 | 232.6 |
|  | -6 | 5.55 | 128 | 43.9 | 79.1 | 23.1 | 292 | 13.1 | 149.8 |
|  | -3 | 5.27 | 121 | 39.1 | 74.3 | 22.9 | 308 | 12.7 | 170.4 |
|  | 1 | 5.53 | 127 | 41.3 | 74.7 | 23.0 | 308 | 12.8 | 192.1 |
|  | 2 | 5.02 | 114 | 37.9 | 75.5 | 22.7 | 301 | 13.1 | 193.8 |
|  | 7 | 4.94 | 112 | 36.7 | 74.3 | 22.6 | 304 | 13.1 | 272.2 |
|  | 14 | 5.22 | 118 | 38.9 | 74.5 | 22.5 | 302 | 12.9 | 250.5 |
|  | 42 | 5.46 | 124 | 40.7 | 74.6 | 22.7 | 305 | 12.3 | 112.3 |

| Treatment  100 mg/kg | Day(s) relative to start date |  |  |  |  |  |  |  |  |
| --- | --- | --- | --- | --- | --- | --- | --- | --- | --- |
|  |  | RBC  (10^12^/liter) | HGB  (g/liter) | HCT  (%) | MCV  (fl) | MCH  (pg) | MCHC  (g/liter) | RDW  (%) | RET  (10^9^/liter) |
| 1509 | -13 | 6.12 | 137 | 46.9 | 76.5 | 22.3 | 291 | 12.6 | 100.6 |
|  | -6 | 6.21 | 136 | 45.9 | 73.9 | 21.8 | 295 | 12.7 | 116.1 |
|  | 1 | 5.89 | 131 | 44.0 | 74.8 | 22.3 | 298 | 12.9 | 152.1 |
|  | 2 | 5.56 | 121 | 42.6 | 76.6 | 21.7 | 284 | 13.1 | 141.4 |
|  | 7 | 5.32 | 115 | 39.3 | 73.8 | 21.6 | 292 | 13.2 | 258.1 |
|  | 14 | 5.94 | 128 | 46.4 | 78.1 | 21.6 | 276 | 12.9 | 220.7 |
|  | 42 | 6.71 | 147 | 51.4 | 76.7 | 21.9 | 285 | 12.6 | 88.2 |

MCV, mean corpuscular volume; MCH, mean corpuscular hemoglobin; MCHC, mean corpuscular hemoglobin concentration; RDW, red cell distribution width; RET, reticulocytes (absolute).
