# Supplementary material for: Developing high-affinity decoy receptors to treat multiple myeloma and diffuse large B cell lymphoma
Source: J Exp Med. 2022 Jul 26;219(9):e20220214. doi: 10.1084/jem.20220214 (PMC9428257; doi:10.1084/jem.20220214)
Supplement: Table S7 — shows male hematology results, part II. [file JEM_20220214_TableS7.docx]

**Table S7.** Male hematology results, part II

| Vehicle control | Day(s) relative to start date |  |  |  |  |  |  |  |
| --- | --- | --- | --- | --- | --- | --- | --- | --- |
|  |  | PLT  (10^9^/liter) | WBC  (10^9^/liter) | NEUT  (10^9^/liter) | LYMP  (10^9^/liter) | MONO  (10^9^/liter) | EOS  (10^9^/liter) | BASO  (10^9^/liter) |
| 1101 | -13 | 257 | 16.07 | 8.51 | 6.91 | 0.24 | 0.17 | 0.11 |
|  | -6 | 297 | 18.52 | 5.88 | 10.99 | 0.47 | 0.83 | 0.16 |
|  | 1 | 295 | 21.84 | 8.67 | 11.78 | 0.45 | 0.60 | 0.14 |
|  | 2 | 270 | 21.24 | 7.36 | 12.58 | 0.41 | 0.49 | 0.18 |
|  | 7 | 327 | 18.12 | 5.08 | 11.85 | 0.43 | 0.50 | 0.11 |
|  | 14 | 386 | 20.46 | 8.21 | 10.86 | 0.43 | 0.63 | 0.16 |
|  | 42 | 280 | 15.70 | 4.30 | 10.26 | 0.38 | 0.47 | 0.12 |

| Treatment  0.1 mg/kg | Day(s) relative to start date |  |  |  |  |  |  |  |
| --- | --- | --- | --- | --- | --- | --- | --- | --- |
|  |  | PLT  (10^9^/liter) | WBC  (10^9^/liter) | NEUT  (10^9^/liter) | LYMP  (10^9^/liter) | MONO  (10^9^/liter) | EOS  (10^9^/liter) | BASO  (10^9^/liter) |
| 1203 | -13 | 347 | 9.14 | 4.36 | 4.32 | 0.30 | 0.07 | 0.04 |
|  | -6 | 436 | 11.74 | 1.82 | 8.93 | 0.49 | 0.30 | 0.11 |
|  | 1 | 387 | 10.69 | 1.43 | 8.52 | 0.26 | 0.34 | 0.07 |
|  | 2 | 369 | 11.81 | 6.25 | 4.95 | 0.37 | 0.14 | 0.05 |
|  | 7 | 410 | 9.25 | 3.78 | 4.95 | 0.24 | 0.19 | 0.04 |
|  | 14 | 436 | 8.93 | 2.54 | 5.80 | 0.27 | 0.25 | 0.03 |
|  | 42 | 364 | 12.90 | 6.83 | 5.37 | 0.38 | 0.19 | 0.07 |

| Treatment  1 mg/kg | Day(s) relative to start date |  |  |  |  |  |  |  |
| --- | --- | --- | --- | --- | --- | --- | --- | --- |
|  |  | PLT  (10^9^/liter) | WBC  (10^9^/liter) | NEUT  (10^9^/liter) | LYMP  (10^9^/liter) | MONO  (10^9^/liter) | EOS  (10^9^/liter) | BASO  (10^9^/liter) |
| 1305 | -13 | 334 | 22.46 | 6.80 | 14.83 | 0.32 | 0.05 | 0.20 |
|  | -6 | 375 | 36.24 | 7.27 | 26.77 | 0.72 | 0.33 | 0.54 |
|  | -3 | 335 | 30.61 | 4.56 | 24.24 | 0.44 | 0.37 | 0.47 |
|  | 1 | 386 | 35.27 | 4.59 | 29.04 | 0.25 | 0.31 | 0.56 |
|  | 2 | 330 | 32.46 | 7.99 | 23.17 | 0.29 | 0.26 | 0.38 |
|  | 7 | 356 | 33.46 | 11.88 | 20.27 | 0.33 | 0.28 | 0.38 |
|  | 14 | 343 | 34.98 | 8.03 | 25.17 | 0.45 | 0.47 | 0.43 |
|  | 42 | 306 | 28.18 | 4.86 | 21.89 | 0.49 | 0.22 | 0.32 |

| Treatment  10 mg/kg | Day(s) relative to start date |  |  |  |  |  |  |  |
| --- | --- | --- | --- | --- | --- | --- | --- | --- |
|  |  | PLT  (10^9^/liter) | WBC  (10^9^/liter) | NEUT  (10^9^/liter) | LYMP  (10^9^/liter) | MONO  (10^9^/liter) | EOS  (10^9^/liter) | BASO  (10^9^/liter) |
| 1407 | -13 | 375 | 14.70 | 4.79 | 9.52 | 0.18 | 0.05 | 0.09 |
|  | -6 | 389 | 30.01 | 13.65 | 14.59 | 1.07 | 0.25 | 0.26 |
|  | -3 | 382 | 16.90 | 1.49 | 14.57 | 0.37 | 0.18 | 0.16 |
|  | 1 | 339 | 19.36 | 1.85 | 16.55 | 0.36 | 0.25 | 0.19 |
|  | 2 | 302 | 17.83 | 4.11 | 12.91 | 0.29 | 0.21 | 0.16 |
|  | 7 | 375 | 16.53 | 4.47 | 11.34 | 0.31 | 0.22 | 0.11 |
|  | 14 | 443 | 26.94 | 5.32 | 20.19 | 0.50 | 0.41 | 0.30 |
|  | 42 | 331 | 22.21 | 2.61 | 18.37 | 0.46 | 0.29 | 0.25 |

| Treatment  100 mg/kg | Day(s) relative to start date |  |  |  |  |  |  |  |
| --- | --- | --- | --- | --- | --- | --- | --- | --- |
|  |  | PLT  (10^9^/liter) | WBC  (10^9^/liter) | NEUT  (10^9^/liter) | LYMP  (10^9^/liter) | MONO  (10^9^/liter) | EOS  (10^9^/liter) | BASO  (10^9^/liter) |
| 1509 | -13 | 452 | 10.38 | 5.67 | 3.82 | 0.70 | 0.03 | 0.09 |
|  | -6 | 494 | 25.43 | 8.46 | 15.40 | 0.65 | 0.25 | 0.36 |
|  | 1 | 515 | 20.92 | 5.45 | 14.11 | 0.58 | 0.37 | 0.21 |
|  | 2 | 457 | 33.35 | 25.95 | 6.15 | 0.93 | 0.10 | 0.15 |
|  | 7 | 409 | 18.22 | 8.40 | 8.97 | 0.47 | 0.13 | 0.14 |
|  | 14 | 599 | 20.36 | 10.09 | 9.39 | 0.45 | 0.19 | 0.15 |
|  | 42 | 510 | 16.62 | 7.42 | 8.33 | 0.52 | 0.14 | 0.12 |

PLT, platelets; WBC, white blood cells, NEUT, neutrophils (absolute); LYMP, lymphocytes (absolute); MONO, monocytes (absolute); EOS, eosinophils (absolute); BASO, basophils.
