# Supplementary material for: Developing high-affinity decoy receptors to treat multiple myeloma and diffuse large B cell lymphoma
Source: J Exp Med. 2022 Jul 26;219(9):e20220214. doi: 10.1084/jem.20220214 (PMC9428257; doi:10.1084/jem.20220214)
Supplement: Table S8 — shows female hematology results, part I. [file JEM_20220214_TableS8.docx]

**Table S8.** Female hematology results, part I

| Vehicle Control | Day(s) relative to start date |  |  |  |  |  |  |  |  |
| --- | --- | --- | --- | --- | --- | --- | --- | --- | --- |
|  |  | RBC  (10^12^/liter) | HGB  (g/liter) | HCT  (%) | MCV  (fl) | MCH  (pg) | MCHC  (g/liter) | RDW  (%) | RET  (10^9^/liter) |
| 2102 | -13 | 6.31 | 141 | 46.3 | 73.4 | 22.4 | 305 | 12.4 | 122.8 |
|  | -6 | 6.75 | 148 | 49.8 | 73.9 | 21.9 | 296 | 11.9 | 114.0 |
|  | 1 | 6.00 | 131 | 44.2 | 73.7 | 21.9 | 297 | 12.0 | 121.5 |
|  | 2 | 5.32 | 116 | 39.0 | 73.3 | 21.7 | 296 | 12.2 | 117.1 |
|  | 7 | 5.45 | 119 | 41.0 | 75.2 | 21.8 | 290 | 12.5 | 255.5 |
|  | 14 | 5.77 | 126 | 42.0 | 72.8 | 21.8 | 300 | 12.2 | 196.7 |
|  | 42 | 6.18 | 136 | 44.3 | 71.7 | 22.1 | 308 | 11.5 | 72.4 |

| Treatment  0.1 mg/kg | Day(s) relative to start date |  |  |  |  |  |  |  |  |
| --- | --- | --- | --- | --- | --- | --- | --- | --- | --- |
|  |  | RBC  (10^12^/liter) | HGB  (g/liter) | HCT  (%) | MCV  (fl) | MCH  (pg) | MCHC  (g/liter) | RDW  (%) | RET  (10^9^/liter) |
| 2204 | -13 | 5.15 | 129 | 42.2 | 82.1 | 25.0 | 305 | 14.4 | 99.3 |
|  | -6 | 5.31 | 136 | 43.1 | 81.1 | 25.5 | 315 | 14.3 | 101.3 |
|  | 1 | 4.90 | 127 | 40.1 | 81.7 | 25.9 | 317 | 14.2 | 95.9 |
|  | 2 | 4.60 | 113 | 37.0 | 80.4 | 24.6 | 306 | 14.4 | 107.8 |
|  | 7 | 4.59 | 114 | 36.9 | 80.5 | 24.8 | 308 | 14.3 | 238.0 |
|  | 14 | 4.95 | 123 | 39.6 | 80.0 | 24.8 | 310 | 14.3 | 180.1 |
|  | 42 | 5.47 | 135 | 43.2 | 79.0 | 24.7 | 313 | 13.6 | 99.0 |

| Treatment  1 mg/kg | Day(s) relative to start date |  |  |  |  |  |  |  |  |
| --- | --- | --- | --- | --- | --- | --- | --- | --- | --- |
|  |  | RBC  (10^12^/liter) | HGB  (g/liter) | HCT  (%) | MCV  (fl) | MCH  (pg) | MCHC  (g/liter) | RDW  (%) | RET  (10^9^/liter) |
| 2306 | -13 | 5.20 | 125 | 40.9 | 78.6 | 24.0 | 306 | 13.1 | 70.8 |
|  | -6 | 5.06 | 123 | 40.1 | 79.2 | 24.4 | 308 | 13.0 | 77.4 |
|  | 1 | 4.98 | 121 | 39.8 | 79.8 | 24.3 | 305 | 13.1 | 80.4 |
|  | 2 | 4.48 | 108 | 35.4 | 78.9 | 24.1 | 305 | 13.3 | 74.3 |
|  | 7 | 4.55 | 111 | 35.8 | 78.6 | 24.3 | 309 | 13.6 | 148.8 |
|  | 14 | 4.97 | 121 | 38.9 | 78.4 | 24.3 | 311 | 13.1 | 97.7 |
|  | 42 | 5.25 | 130 | 40.1 | 76.4 | 24.7 | 323 | 12.2 | 40.1 |

| Treatment  10 mg/kg | Day(s) relative to start date |  |  |  |  |  |  |  |  |
| --- | --- | --- | --- | --- | --- | --- | --- | --- | --- |
|  |  | RBC  (10^12^/liter) | HGB  (g/liter) | HCT  (%) | MCV  (fl) | MCH  (pg) | MCHC  (g/liter) | RDW  (%) | RET  (10^9^/liter) |
| 2408 | -13 | 5.23 | 128 | 40.9 | 78.3 | 24.4 | 312 | 12.1 | 77.8 |
|  | -6 | 5.30 | 129 | 42.4 | 80.1 | 24.3 | 303 | 11.8 | 90.7 |
|  | 1 | 5.08 | 126 | 39.7 | 78.2 | 24.9 | 318 | 12.2 | 84.3 |
|  | 2 | 4.70 | 115 | 37.1 | 79.0 | 24.5 | 310 | 12.2 | 97.1 |
|  | 7 | 4.50 | 109 | 34.8 | 77.4 | 24.3 | 314 | 12.4 | 177.5 |
|  | 14 | 4.85 | 117 | 37.0 | 76.2 | 24.1 | 316 | 11.9 | 91.4 |
|  | 42 | 5.28 | 131 | 39.7 | 75.2 | 24.7 | 329 | 11.5 | 46.0 |

| Treatment  100 mg/kg | Day(s) relative to start date |  |  |  |  |  |  |  |  |
| --- | --- | --- | --- | --- | --- | --- | --- | --- | --- |
|  |  | RBC  (10^12^/liter) | HGB  (g/liter) | HCT  (%) | MCV  (fl) | MCH  (pg) | MCHC  (g/liter) | RDW  (%) | RET  (10^9^/liter) |
| 2510 | -13 | 5.25 | 112 | 37.9 | 72.3 | 21.3 | 295 | 14.6 | 152.9 |
|  | -6 | 5.42 | 113 | 38.9 | 71.8 | 20.9 | 291 | 14.0 | 141.6 |
|  | 1 | 5.18 | 109 | 37.0 | 71.6 | 21.1 | 295 | 14.1 | 143.2 |
|  | 2 | 4.67 | 97 | 34.5 | 73.9 | 20.8 | 282 | 14.1 | 141.3 |
|  | 7 | 4.84 | 103 | 34.0 | 70.2 | 21.2 | 302 | 14.5 | 194.5 |
|  | 14 | 5.10 | 108 | 36.5 | 71.5 | 21.1 | 295 | 14.1 | 197.4 |
|  | 42 | 5.40 | 114 | 36.9 | 68.3 | 21.1 | 309 | 12.9 | 100.6 |

MCV, mean corpuscular volume; MCH, mean corpuscular hemoglobin; MCHC, mean corpuscular hemoglobin concentration; RDW, red cell distribution width; RET, reticulocytes (absolute).
