# Supplementary material for: Developing high-affinity decoy receptors to treat multiple myeloma and diffuse large B cell lymphoma
Source: J Exp Med. 2022 Jul 26;219(9):e20220214. doi: 10.1084/jem.20220214 (PMC9428257; doi:10.1084/jem.20220214)
Supplement: Table S9 — shows female hematology results, part II. [file JEM_20220214_TableS9.docx]

**Table S9.** Female hematology results, part II

| Vehicle Control | Day(s) relative to start date |  |  |  |  |  |  |  |
| --- | --- | --- | --- | --- | --- | --- | --- | --- |
|  |  | PLT  (10^9^/liter) | WBC  (10^9^/liter) | NEUT  (10^9^/liter) | LYMP  (10^9^/liter) | MONO  (10^9^/liter) | EOS  (10^9^/liter) | BASO  (10^9^/liter) |
| 2102 | -13 | 409 | 12.30 | 8.25 | 3.64 | 0.28 | 0.01 | 0.06 |
|  | -6 | 465 | 17.31 | 7.58 | 8.49 | 0.71 | 0.26 | 0.13 |
|  | 1 | 463 | 17.06 | 6.68 | 9.41 | 0.47 | 0.21 | 0.13 |
|  | 2 | 401 | 19.30 | 10.82 | 7.45 | 0.65 | 0.16 | 0.09 |
|  | 7 | 466 | 18.84 | 10.43 | 7.52 | 0.53 | 0.14 | 0.10 |
|  | 14 | 498 | 22.49 | 11.69 | 9.34 | 0.80 | 0.30 | 0.17 |
|  | 42 | 406 | 15.61 | 6.94 | 7.72 | 0.55 | 0.18 | 0.11 |

| Treatment  0.1 mg/kg | Day(s) relative to start date |  |  |  |  |  |  |  |
| --- | --- | --- | --- | --- | --- | --- | --- | --- |
|  |  | PLT  (10^9^/liter) | WBC  (10^9^/liter) | NEUT  (10^9^/liter) | LYMP  (10^9^/liter) | MONO  (10^9^/liter) | EOS  (10^9^/liter) | BASO  (10^9^/liter) |
| 2204 | -13 | 291 | 10.33 | 5.08 | 4.84 | 0.29 | 0.02 | 0.05 |
|  | -6 | 336 | 17.92 | 4.68 | 11.78 | 0.85 | 0.26 | 0.19 |
|  | 1 | 314 | 15.43 | 4.61 | 9.67 | 0.66 | 0.24 | 0.13 |
|  | 2 | 299 | 12.04 | 5.17 | 6.24 | 0.33 | 0.15 | 0.07 |
|  | 7 | 371 | 12.08 | 5.61 | 5.78 | 0.43 | 0.11 | 0.08 |
|  | 14 | 343 | 15.24 | 5.98 | 8.20 | 0.66 | 0.18 | 0.11 |
|  | 42 | 292 | 13.39 | 4.75 | 7.79 | 0.47 | 0.19 | 0.08 |

| Treatment  1 mg/kg | Day(s) relative to start date |  |  |  |  |  |  |  |
| --- | --- | --- | --- | --- | --- | --- | --- | --- |
|  |  | PLT  (10^9^/liter) | WBC  (10^9^/liter) | NEUT  (10^9^/liter) | LYMP  (10^9^/liter) | MONO  (10^9^/liter) | EOS  (10^9^/liter) | BASO  (10^9^/liter) |
| 2306 | -13 | 511 | 18.73 | 12.88 | 5.48 | 0.23 | 0.04 | 0.06 |
|  | -6 | 459 | 19.87 | 6.05 | 12.81 | 0.52 | 0.19 | 0.17 |
|  | 1 | 551 | 16.10 | 3.61 | 11.82 | 0.26 | 0.21 | 0.10 |
|  | 2 | 464 | 22.11 | 11.37 | 10.04 | 0.38 | 0.13 | 0.11 |
|  | 7 | 468 | 16.55 | 7.28 | 8.71 | 0.32 | 0.10 | 0.08 |
|  | 14 | 462 | 19.52 | 6.17 | 12.31 | 0.47 | 0.31 | 0.15 |
|  | 42 | 426 | 19.46 | 6.04 | 12.49 | 0.39 | 0.28 | 0.16 |

| Treatment  10 mg/kg | Day(s) relative to start date |  |  |  |  |  |  |  |
| --- | --- | --- | --- | --- | --- | --- | --- | --- |
|  |  | PLT  (10^9^/liter) | WBC  (10^9^/liter) | NEUT  (10^9^/liter) | LYMP  (10^9^/liter) | MONO  (10^9^/liter) | EOS  (10^9^/liter) | BASO  (10^9^/liter) |
| 2408 | -13 | 283 | 11.59 | 4.41 | 6.73 | 0.30 | 0.02 | 0.05 |
|  | -6 | 304 | 17.57 | 6.30 | 10.31 | 0.44 | 0.20 | 0.17 |
|  | 1 | 324 | 14.88 | 3.99 | 9.90 | 0.42 | 0.33 | 0.10 |
|  | 2 | 297 | 18.24 | 7.51 | 9.92 | 0.42 | 0.17 | 0.10 |
|  | 7 | 294 | 17.57 | 10.20 | 6.80 | 0.32 | 0.10 | 0.08 |
|  | 14 | 307 | 17.15 | 5.12 | 10.95 | 0.58 | 0.29 | 0.10 |
|  | 42 | 300 | 21.70 | 9.11 | 11.29 | 0.74 | 0.22 | 0.15 |

| Treatment  100 mg/kg | Day(s) relative to start date |  |  |  |  |  |  |  |
| --- | --- | --- | --- | --- | --- | --- | --- | --- |
|  |  | PLT  (10^9^/liter) | WBC  (10^9^/liter) | NEUT  (10^9^/liter) | LYMP  (10^9^/liter) | MONO  (10^9^/liter) | EOS  (10^9^/liter) | BASO  (10^9^/liter) |
| 2510 | -13 | 359 | 13.64 | 7.64 | 5.45 | 0.36 | 0.07 | 0.06 |
|  | -6 | 452 | 21.25 | 8.50 | 11.41 | 0.55 | 0.51 | 0.14 |
|  | 1 | 418 | 18.34 | 6.48 | 10.88 | 0.40 | 0.38 | 0.09 |
|  | 2 | 389 | 19.60 | 10.71 | 8.31 | 0.28 | 0.13 | 0.08 |
|  | 7 | 434 | 18.73 | 13.53 | 4.70 | 0.22 | 0.17 | 0.04 |
|  | 14 | 433 | 20.69 | 10.11 | 9.67 | 0.31 | 0.35 | 0.11 |
|  | 42 | 412 | 20.23 | 12.52 | 6.91 | 0.35 | 0.28 | 0.11 |

PLT, platelets, NEUT, neutrophils (absolute); LYMP, lymphocytes (absolute); MONO, monocytes (absolute); EOS, eosinophils (absolute); BASO, basophils (absolute).
